# Synthesis and Antibacterial Activity Studies of the Conjugates of Curcumin with *closo*-Dodecaborate and Cobalt Bis(Dicarbollide) Boron Clusters †

**DOI:** 10.3390/molecules27092920

**Published:** 2022-05-03

**Authors:** Anna A. Druzina, Natalia E. Grammatikova, Olga B. Zhidkova, Natalia A. Nekrasova, Nadezhda V. Dudarova, Irina D. Kosenko, Mikhail A. Grin, Vladimir I. Bregadze

**Affiliations:** 1 A.N. Nesmeyanov Institute of Organoelement Compounds, Russian Academy of Sciences, 28 Vavilov Str., 119991 Moscow, Russia; zolga57@mail.ru (O.B.Z.); nekrasova_na@list.ru (N.A.N.); nadezjdino_96@mail.ru (N.V.D.); kosenko@ineos.ac.ru (I.D.K.); bre@ineos.ac.ru (V.I.B.); 2 G.F. Gause Institute of New Antibiotics, 11 B. Pirogovskaya, 119021 Moscow, Russia; ngrammatikova@yandex.ru; 3 M.V. Lomonosov Institute of Fine Chemical Technology, MIREA—Russian Technological University, 86 Vernadsky Av., 119571 Moscow, Russia; michael_grin@mail.ru

**Keywords:** polyhedral boron hydrides, *closo*-dodecaborate, cobalt bis(dicarbollide), curcumin, “click” reaction, antibacterial activity, *Staphylococcus aureus*, *Enterococcus faecalis*

## Abstract

A series of novel conjugates of cobalt bis(dicarbollide) and *closo*-dodecaborate with curcumin were synthesized by copper(I)-catalyzed azide-alkyne cycloaddition. These conjugates were tested for antibacterial activity. It was shown that all derivatives are active when exposed to *Bacillus cereus* ATCC 10702 and are not active against Gram-negative microorganisms and *Candida albicans* at the maximum studied concentration of 1000 mg/L. The conjugate of alkynyl-curcumin with azide synthesized from the tetrahydropyran derivative of cobalt bis(dicarbollide) exhibited activity against Gram-positive microorganisms: *Staphylococcus aureus* ATCC 29213, *Enterococcus faecalis* ATCC 29212 and the clinical isolate MRSA 17, that surpassed curcumin by 2–4 times.

## 1. Introduction

The constant interest in polyhedral boron hydrides (borohydride clusters and metallacarboranes) has provided excellent contributions in chemistry of organoelement compounds during the past decades [1,2,3]. The presence of hydride BH vertices and charge delocalization over the whole structure confers a chemical stability [3,4], resistance to catabolism [5], and amphiphilicity [6,7] to boron clusters. Apart from the interest in their electronic properties, burgeoning research efforts have been dedicated to polyhedral boron hydride application in medicinal chemistry [8]. The possibility of the formation of anionic compounds is very important from the point of view of physiology, since it allows the synthesis of alkali and alkaline earth salts of the target compounds, which are highly soluble in water and possess low toxicity. It was established that stable boron cluster compounds and organic compounds have a tendency to self-assemble in an aqueous solution [7,9] and interact with components of biological systems, such as lipid membranes [10,11,12,13] and proteins [9,13,14,15], through different mechanisms, which also opens up good prospects for creation of drugs based on them.

Studies on medicinal applications of polyhedral boron hydrides have mostly focused on agents for boron neutron capture therapy (BNCT) [16,17], contrast agents for MRI diagnostics [18] and compounds with antiviral activity [19,20]. However, their antimicrobial properties have been investigated only to a limited degree [21]. Although the potential application of boron clusters as building blocks for novel antimicrobials has been hypothesized as early as the 1980s [22]. It was shown that the boron clusters have the potential to become new chemical leads in antibacterial therapy because their derivatives show promising antibacterial activity and low sensitivity to both genetic and phenotypic mechanisms of pathogen resistance.

The examples of boron-containing derivatives that can effectively act directly on a bacterium are either low-molecular-weight boron cluster derivatives or biomolecules based on them. It is known that derivatives of *nido*-carboranes, containing lipophilic alkyl groups, display the best antimicrobial activity against Gram-positive bacteria (*S. aureus*, *S. pyogenes*) [23]. The cobalt bis(dicarbollide) derivatives that have shown antibacterial activity against methicillin-resistant *P. aeruginosa* contained simple organic compounds attached through ether linkers [24,25,26], whereas cobalt bis(dicarbollide) derivatives containing primary, secondary, tertiary, and quaternary amines were obtained with antimicrobial activity against Gram-positive bacteria (*S. epidermidis*, *S. aureus* and *E. faecalis*), and against Gram-negative bacteria (two strains of *P. aeruginosa* and *E. coli*) [27,28]. Analogues, in which the ethylenediamine linker is replaced by oxa-, thia- or heterocyclic groups containing *o*-carborane, showed activity against the Gram-negative bacteria *E. coli* [29]. Other examples of compounds based on cluster boron anions exhibiting antibacterial activity are derivatives based on carboranyl phosphonates [30,31]. A series of *closo*-dodecaborate amides and diboraoxazoles derivatives showed high and specific activity against Gram-negative (*N. gonorrhoeae*) but low activity against the Gram-positive (*S. aureus* and *E. faecalis*) bacteria [32].

Some examples of conjugates of polyhedral boron hydrides with various biomolecules are presented in the literature and their antibacterial activity has been studied. O-carboranylalanine showed high activity against all asexual spore forms of *P. halstedii* [33]. Ferrocene-*o*-carborane derivatives were tested against the Gram-positive *S. aureus* and the Gram-negative *K. pneumoniae*, *A. baumannii*, *P. mirabilis* and *E. coli* [34,35]. An *o*-carborane derivative containing two ferrocene moieties showed antibacterial activity against two clinical isolates of MDR bacteria: Gram-positive *S. aureus* and Gram-negative *P. aeruginosa* [36]. The thymidine derivatives containing one or two *o*-carborane clusters displayed the best activity against *M. smegmatis* [37]. Penicillin analogs were investigated, wherein the phenyl ring was substituted by *o*-carborane, *m*-carborane or *p*-carborane clusters, and showed activity against both methicillin-sensitive and -resistant *S. aureus* [38]. The most active analog contained *p*-carborane, followed by those with *m*-carborane and *o*-carborane. Thus, the order of antibacterial activity of the analogs is associated with the order of lipophilicity of the attached carborane, that is, the most active analog is that which contains the most lipophilic *p*-carborane.

Importantly, the choice of organic derivatives and natural biomolecules attached to the cluster vertices proves vital in changing the functionality and antimicrobial activity of the product boron cluster antimicrobials. In this contribution we present the synthesis of novel conjugates of polyhedral boron hydrides (*closo*-dodecaborate anion and cobalt bis(dicarbollide)) with curcumin and study of their antibacterial activity.

## 2. Results and Discussion

### 2.1. Synthesis of the Conjugates of Curcumin with closo-Dodecaborate and Cobalt Bis(Bicarbollide) Boron Clusters

Curcumin is an herbal supplement originating from turmeric (root of the rhizome Curcuma longa) and belongs to the curcuminoids group, which are plant phenol metabolites showing a wide range of pharmacological activities [39,40]. Curcumin has shown strong antibacterial potency against some Gram-positive and Gram-negative bacteria by damaging their membranes [41,42,43]. The consistently growing demand for potent compounds for drug discovery has given birth to simple and efficient synthetic routes for creating libraries of biologically active molecules. Among the methods for obtaining bioconjugates, the Cu(I)-catalyzed 1,3-dipolar [3 + 2] cycloaddition reaction of alkynes to azides is widely used, leading to the formation of 1,2,3 triazoles, termed as the “click”-reaction” [44,45,46]. Earlier, the “click”-reaction was successfully used to obtain a wide range of conjugates of polyhedral boron hydrides with various biologically active molecules, such as nucleosides [47] and chlorine e*_6_* [48], as well as derivatives of cholesterol based on cobalt/iron bis(dicarbollide) [49,50,51,52], *closo*-dodecaborate dianion [53] and *nido*-carborane [54,55,56]. Such reactions must proceed rapidly under ambient conditions, resulting in a high yield of desired 1,2,3-triazole. In the present work, we used the “click” methodology to obtain new conjugates of the cobalt bis(dicarbollide) and *closo*-dodecaborate with curcumin. At the time of our study, the only example of boron-containing curcumin presented in the literature is its spiro borate ether [57], but the conjugates of curcumin with boron clusters are not known.

**Scheme 1:**
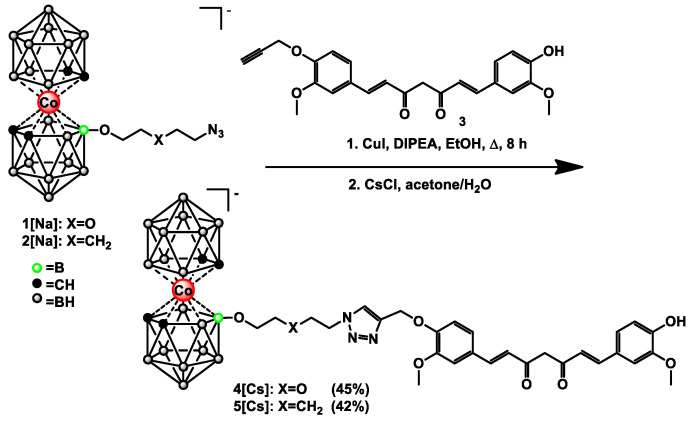
Synthesis of conjugates of cobalt bis(dicarbollide) with curcumin **4** and **5**.

Thus, as the first goal of our investigation, we prepared the azido derivatives based on the cobalt bis(dicarbollide) **1** and **2** [47,58] and *closo*-dodecaborate **6**–**8** [53,59,60] by the nucleophilic ring-opening reactions of oxonium derivatives of boron clusters with NaN_3_ and alkynyl curcumine [61] according to the known procedures. Further it was found that azides synthesized from 1,4-dioxane and tetrahydropyran derivatives of cobalt bis(dicarbollide) **1** and **2** readily undergo “click” reactions with alkynyl curcumin **3** in the presence of a CuI catalyst and diisopropylethylamine (DIPEA) as a base in ethanol upon prolonged reflux during 8 h to give novel boron conjugates **4** and **5,** which were isolated in a form of cesium salts with good yields (42–45%) (Scheme 1). Novel anionic boron conjugates with curcumin **4** and **5** were isolated in a form of cesium salts.

The structures of the compounds **4** and **5** were established by ^1^H-, ^11^B- and ^13^C-NMR, IR-spectroscopy and high-resolution mass-spectrometry (see Supplementary Materials). In the ^1^H-NMR spectra of the obtained conjugates, the characteristic signals of the triazole CH hydrogens appear in the region of 8.06–8.16 ppm. In the ^13^C-NMR spectra, the signals of the triazole CH carbons for **4** and **5** are observed in the range of 121.5–122.2 ppm, whereas the signals of the triazole C carbons appear at 140.1 ppm. In the ^1^H-NMR spectra, the characteristic signals of the CH_3_ groups of curcumin were observed at 3.88 and 3.94 ppm for compounds **4** and **5**. The ^13^C-NMR spectra of compounds **4** and **5** display the characteristic signals of -C=O carbons of the curcumin skeleton in the region at 183.2–183.9 ppm. In addition, the signals of the CH_carb_ groups in the ^1^H-NMR spectra appear as broad singlets at 4.22 ppm for conjugate **4** and 4.27 and 4.20 ppm for conjugate **5**; in the ^13^C-NMR spectra, the signals of CH_carb_ groups appeared in the range 46.5–53.9 ppm. The IR spectra of compounds **4** and **5** exhibit absorption bands characteristic of the BH groups 2543–2554 cm^−1^, the 1,2,3-triazole rings 1520–1523 cm^−1^ and C=O groups 1597–1638 cm^−1^. The mass spectra of the synthesized conjugates showed characteristic peaks with the cobalt bis(dicarbollide) isotope pattern centered at 859.4824 and 857.5024, corresponding to the molecular ions of compounds **4** and **5**, respectively.

**Scheme 2:**
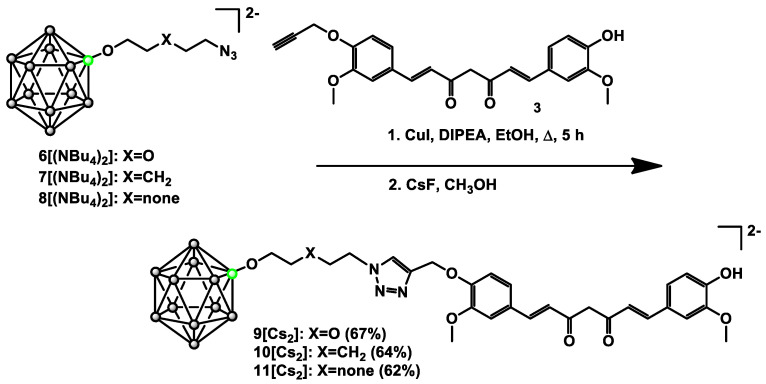
Synthesis of conjugates of *closo*-dodecaborate with curcumin **9**–**11**.

The “click”-reaction of azides prepared from 1,4-dioxane, tetrahydropyran and tetrahydrofuran derivatives of *closo*-dodecaborate **6**–**8** with alkynyl-curcumin **3** after 5 h led to dianionic **9**–**11** in a form of cesium salts with good yields (62–67% yield) (Scheme 2).

The structures of the compounds **9**–**11** were established by ^1^H-, ^11^B- and ^13^C-NMR, IR-spectroscopy and high-resolution mass-spectrometry (see Supplementary Materials). The ^1^H-NMR spectra of complexes **9**–**11** contained signals for the protons of the triazole ring at 8.26–8.38 ppm. In the ^13^C-NMR spectra, the signals of the triazole CH carbons for **9**–**11** were observed in the range of 122.2–122.9 ppm, whereas the signals of the triazole C carbons appeared in the region of 140.2–140.8 ppm. In the ^1^H-NMR spectra, the characteristic signals of the CH_3_ groups of curcumin were observed in the region of 3.78–3.82 ppm for conjugates **9**–**11**. The ^13^C-NMR spectra of compounds **9**–**11** displayed the characteristic signals of -C=O carbons of the curcumin in the region at 182.6–184.4 ppm. In the ^11^B-NMR spectrum of compounds **9**–**11**, the signal for the B-O atom, as expected, presented at 6.3–6.5 ppm. The IR spectra of compounds **9**–**11** exhibited absorption bands characteristic of the BH groups 2478–2492 cm^−1^, the 1,2,3-triazole rings 1511–1514 cm^−1^ and C=O groups 1597–1655 cm^−1^. The mass spectra of the synthesized conjugates showed characteristic peaks with the *closo*-dodecaborate isotope pattern centered at 810.3132, 808.3347, and 794.3205, corresponding to the molecular ions of compounds **9**–**11**, respectively.

### 2.2. Antibacterial Activity Studies

Studying the biological activity of new curcumin derivatives is important for analyzing the relationship between structure and function in order to understand the strategy for creating highly active compounds. Our studies, the results of which are presented in Table 1, showed that none of the compounds showed activity against Gram-negative microorganisms in the range of concentrations studied. Differences in MIC values were found for Gram-positive bacteria. *Bacillus cereus* ATCC 10702 was susceptible to all samples. The strains of *S. aureus* ATCC 29213, MRSA 17, and *E. faecalis* ATCC 29212 showed greater sensitivity to derivative **5**. However, the effect of compound **5** on the reference culture of *S. aureus* ATCC 29213 and *B. cereus* ATCC 10702 was similar to that of curcumin. With respect to *E. faecalis* ATCC 29212, none of the samples showed activity at the studied concentrations (MIC > 1000 mg/L), with the exception of compound **5** (MIC 250 mg/L).

In relation to fungal cultures, growth inhibition of *A. fumigatus* ATCC 46645 was detected only in the presence of sample **5**, for the remaining samples, MIC > 1000 mg/L. Complete growth inhibition of clinical isolates of *C. albicans* was not observed in the presence of all test compounds. However, a similar decrease in growth density compared to the untreated control was found for curcumin and derivatives **4** and **5** in the concentration range from 1000 mg/L to 15.6 mg/L.

Based on the data obtained, it can be concluded that the samples are distributed according to activity in the following order: 1. The derivative **5** (according to MIC values for Gram-positive bacteria, *A. fumigatus*, a decrease in the growth density of *C. albicans*) 2. Curcumin (according to MIC values for Gram-positive bacteria) bacteria, reduced growth density of *C. albicans*), 3. The compound **4** (according to MIC values for Gram-positive bacteria, reduced growth density of *C. albicans*), 4. The conjugates **9**–**11** (equally, active only when exposed to *Bacillus cereus* ATCC 10702).

## 3. Materials and Methods

### 3.1. General Methods

The azido derivatives of cobalt bis(dicarbollide) (8-[N_3_-(O(CH_2_)_2_)_2_]-3,3′-Co(1,2-C_2_B_9_H_10_)(1′,2′-C_2_B_9_H_11_)Na **1** [47] (8-[N_3_-(CH_2_)_5_O]-3,3′-Co(1,2-C_2_B_9_H_10_)(1′,2′-C_2_B_9_H_11_))Na **2** [58], the azido derivatives of *closo*-dodecaborate [B_12_H_11_-(O(CH_2_)_2_)_2_N_3_](NBu_4_)_2_ **6** [59,60], [B_12_H_11_O(CH_2_)_5_N_3_][(NBu_4_)_2_] **7** [53], [B_12_H_11_O(CH_2_)_4_N_3_](NBu_4_)_2_ **8** [59], alkynyl-curcumin **3** [61] were prepared according to the literature. Curcumin (Acros Organoics, Loughborough, U.K.), diisopropylethylamine (Carl Roth GmbH, Karlsruhe, Germany), CuI (PANREAC QUIMICA SA, Barcelona, Spain), were used without further purification. DMF, ethanol, CH_3_CN, CH_2_Cl_2_ and NaN_3_, propargyl bromide, 80 wt% solution in toluene (Acros Organics, Loughborough, UK) were commercially analytical grade reagents. The reaction progress was monitored by thin-layer chromatography (Merck F245 silica gel on aluminum plates). Acros Organics silica gel (0.060–0.200 mm) was used for column chromatography. The NMR spectra at 400.1 MHz (^1^H), 128.4 MHz (^11^B) and 100.0 MHz (^13^C) were recorded with a Bruker Avance-400 spectrometer (Bruker, KarlsruheZurich, Switzerland-Germany). The residual signal of the NMR solvent relative to Me_4_Si was taken as the internal reference for ^1^H- and ^13^C-NMR spectra. ^11^B-NMR spectra were reference using BF_3_∙Et_2_O as external standard. Infrared spectra were recorded on Spectra SF 2000 (OKB SPECTRUM, Saint-Petersburg, Russia) instrument. High resolution mass spectra (HRMS) were measured on a mictOTOF II (Bruker Daltonic, Bremen, Germany) instrument using electrospray ionization (ESI). The measurements were done in a negative ion mode (interface capillary voltage 3200 V); mass range from *m/z* 50 to *m/z* 3000; external or internal calibration was done with ESI Tuning Mix, Agilent. A syringe injection was used for solutions in acetonitrile (flow rate 3 µL/min). Nitrogen was applied as a dry gas; interface temperature was set at 180 °C.

#### General Procedure for the Synthesis of the Conjugates of Cobalt Bis(Dicarbollide) with Curcumin **3** and **4**

A mixture of alkynyl-curcumin **3** (1 eq.), azido derivatives of cobalt bis(dicarbolldie) **1** or **2** (1 eq.), diisopropylethylamine (0.5–1 mL) and CuI (0.1 eq.) in 10–20 mL ethanol was heated under reflux for 8 h. Then the reaction mixture was cooled to room temperature and was passed through ca. 2–3 cm of silica. The system was washed with EtOH until the product ceased to be detected by thin layer chromatography. Then solvent was removed in vacuo. The residue was quenched into ethyl acetate (100 mL). The resulting mixture was washed with 1M HCl (4 × 50 mL) and dried (Na_2_SO_4_). Then the ethyl acetate was evaporated. The residue was dissolved in 5 mL of acetone. To the resulting solution, 1 g of CsCl in 100 mL of water was added. The crude product was purified on a silica column using CH_2_Cl_2_-CH_3_CN as an eluent to give the desired products **4** and **5**.

### 3.2. Synthesis of (8-[(H(CH_2_[COCH=CH(OCH_3_)C_6_H_3_O]_2_))-CH_2_-C-CH-N_3_((CH_2_)_2_O)_2_]-3,3′-Co(1,2-C_2_B_9_H_10_)(1′,2′-C_2_B_9_H_11_))Cs ***4***

Prepared from compound **1** (0.14 g, 0.30 mmol), alkynyl-curcumin **3** (0.12 g, 0.30 mmol), diisopropylethylamine (1 mL, 0.74 g, 5.73 mmol) and CuI (0.006 g, 0.03 mmol) in 20 mL of ethanol. The product was obtained as a a white solid of **4** (0.13 g, yield 45%). ^1^H-NMR (400 MHz, acetone-*d*_6_): δ 16.41 (1H, br.s, O*H*), 8.16 (1H, s, -C*H*CN_3_), 7.62 (2H, d, 2×-C*H*=CH-, *J* = 18.0 Hz), 7.35 (2H, d, 2×-C*H*=C *in phehyl, J* = 8.2 Hz), 7.24 (4H, m, 2×-C*H*=C *in phehyl*, 2×-CH=C*H*-), 6.90 (1H, d, C=C*H- in phehyl*, *J* = 8.1 Hz), 6.76 (1H, m, C=C*H- in phehyl*), 6.02 (1H, s, =C*H*-C), 5.28 (2H, s, OC*H_2_-*C), 4.61 (2H, m, BOC*H*_2_), 4.22 (4H, br.s, C*H_carb_*), 3.94 (3H, s, C*H_3_*OC_6_H_3_-), 3.88 (3H, s, C*H_3_*OC_6_H_3_-), 3.62 (2H, m, -C*H_2_*O), 3.54 (4H, m, -OC*H_2_*, -C*H_2_*N_3_), 1.5–0.5 (br.m, BH) ppm; ^11^B-NMR (128 MHz, acetone-*d*_6_): δ 23.1 (1B, s), 4.4 (1B, d, *J* = 136 Hz), 0.5 (1B, d, *J* = 151), −2.3 (1B, d, *J* = 142 Hz), −4.3 (2B, d, *J* = 153 Hz), −7.2 (2B, d, *J* = 128 Hz), −8.0 (4B, d, *J* = 118 Hz), −17.2 (2B, d, *J* = 151 Hz), −20.3 (2B, d, *J* = 160 Hz), −22.0 (1B, d, *J* unsolved), −28.3 (1B, d, *J* = 173 Hz) ppm; ^13^C-NMR (101 MHz, acetone-*d*_6_): 183.9 (-C=O), 183.3 (-C=O), 150.3 (=*C*-O-CH_3_ *in phehyl*), 150.1 (=*C*-O-CH_3_ *in phehyl*), 149.2 (=*C*-O-CH_2_- *in phehyl*), 147.9 (=*C*-OH *in phehyl*), 142.8 (-*C*H=CH-), 140.6 (-*C*H=CH-), 140.1 (*C*N_3_CH), 139.0 (=*C*H-C=O), 129.7 (=*C*H-C=O), 125.0 (=*C*-CH- *in phehyl*), 123.1 (-*C*=CH- *in phehyl*), 122.4 (=*C*H-CH- *in phehyl*), 122.2 (CN_3_*C*H), 121.5 (-*C*H=CH- *in phehyl*), 115.3 (=*C*H-C-O-CH_3_), 113.8 (-*C*H=C-O-CH_3_), 110.8 (-*C*H=C-OH), 110.5 (=*C*H-C-O-CH_2_-), 100.9 (O*C*H_2_-), 71.8 (*C*H_2_O-), 69.2 (O*C*H_2_-), 68.5 (O*C*H_2_-), 62.2 (O-CH_3_), 55.4 (O-CH_3_), 55.3 (*C*H_2_-C=O), 53.9 (*C*H_2_N), 49.9 (*C*H_carb_), 46.5 (*C*H_carb_) ppm. HRMS (ESI) *m/z* for [C_32_H_51_B_18_CoN_3_O_8_]^-^ calcd 859.4820, found: 859.4824.

### 3.3. Synthesis of (8-[(H(CH_2_[COCH=CH(OCH_3_)C_6_H_3_O]_2_))-CH_2_-C-CH-N_3_(CH_2_)_5_O]-3,3′-Co(1,2-C_2_B_9_H_10_)(1′,2′-C_2_B_9_H_11_))Cs ***5***

Prepared from compound **2** (0.15 g, 0.32 mmol), alkynyl-curcumin **3** (0.13 g, 0.32 mmol), diisopropylethylamine (1 mL, 0.74 g, 5.73 mmol) and CuI (0.006 g, 0.03 mmol) in 20 mL of ethanol. The product was obtained as a a white solid of **5** (0.13 g, yield 42%). ^1^H-NMR (400 MHz, acetone-*d*_6_): δ 16.40 (1H, br.s, OH), 8.08 (1H, s, -C*H*CN_3_), 7.63 (2H, d, 2×-C*H*=CH-, *J* = 16.2 Hz), 7.35 (2H, d, 2×-C*H*=C *in phehyl, J* = 6.4 Hz), 7.23 (4H, m, 2×-C*H*=C *in phehyl*, 2×-CH=C*H*-), 6.90 (1H, d, -C=C*H- in phehyl*, *J* = 8.1 Hz), 6.76 (1H, dd, C=C*H- in phehyl*, *J* = 15.8, 13.6 Hz), 6.02 (1H, s, =C*H*-C), 5.27 (2H, s, OC*H_2_-*C), 4.44 (2H, t, BOC*H*_2_-, *J* = 7.0 Hz), 4.27 (2H, s, C*H_carb_*), 4.20 (2H, s, C*H_carb_*), 3.94 (3H, s, C*H_3_*OC_6_H_3_-), 3.88 (3H, s, C*H_3_*OC_6_H_3_-), 3.47 (2H, t, C*H_2_*-, 6.1 Hz), 1.94 (2H, m, -C*H_2_*), 1.53 (2H, m, -C*H_2_*CH*_2_*N_3_), 1.41 (2H, m, -CH_2_C*H_2_*N_3_), 1.5–0.5 (br.m, BH) ppm; ^11^B-NMR (128 MHz, acetone-*d*_6_): 22.9 (1B, s, B(8)-O), 3.7 (1B, d, *J* =123 Hz), 0.2 (1B, d, *J* = 156 Hz), −2.4 (1B, d, *J* = 140 Hz), −4.3 (1B, d, *J* = 153 Hz), −7.5 (3B, d, *J* = 118 Hz), −8.3 (4B, d, *J* = 113 Hz), −17.3 (2B, d, *J* = 165 Hz), −20.4 (2B, d, *J* = 156 Hz), −22.0 (1B, d, *J* = 156 Hz), −28.6 (1B, d, *J* = 173 Hz) ppm; ^13^C-NMR (101 MHz, acetone-*d*_6_): 183.9 (-C=O), 183.2 (-C=O), 150.2 (=*C*-O-CH_3_ *in phehyl*), 150.1(=*C*-O-CH_3_ *in phehyl*), 149.1(=*C*-O-CH_2_- *in phehyl*), 149.0 (=*C*-OH *in phehyl*), 147.9 (-*C*H=CH-), 140.6 (-*C*H=CH-), 140.1 (*C*N_3_CH), 128.6 (=*C*H-C=O), 127.3 (=*C*H-C=O), 123.1 (=*C*-CH- *in phehyl*), 122.4 (-*C*=CH- *in phehyl*), 122.2 (=*C*H-CH- *in phehyl*), 121.5 (CN_3_*C*H), 121.7 (-*C*H=CH- *in phehyl*), 115.3 (=*C*H-C-O-CH_3_), 113.8 (-*C*H=C-O-CH_3_), 110.8 (-*C*H=C-OH), 110.5 (=*C*H-C-O-CH_2_-), 100.9 (-O-*C*H_2_-), 71.8 (*C*H_2_O), 69.1 (O-CH_3_), 68.5 (O-CH_3_), 55.4 (*C*H_2_-C=O), 55.3 (*C*H_2_N), 55.2 (*C*H_2_), 53.9 (*C*H_carb_), 46.5 (*C*H_carb_), 43.1 (*C*H_2_), 43.0 (*C*H_2_) pmm. HRMS (ESI) *m/z* for [C_33_H_53_B_18_CoN_3_O_7_]^-^ calcd 857.5028, found: 857.5024.

#### General Procedure for the Synthesis of the Conjugates of c*loso*-Dodecaborate with Curcumin **9**–**11**

A mixture of alkynyl-curcumin **3** (1 eq.), azido derivatives of *closo*-dodecaborate **6**–**8** (1 eq.), diisopropylethylamine (0.5–1 mL) and CuI (0.1 eq.) in 10–20 mL ethanol was heated under reflux for 5 h. Then the reaction mixture was cooled to room temperature and was passed through ca. 2–3 cm of silica. The system was washed with EtOH until the product ceased to be detected by thin layer chromatography. Then solvent was removed in vacuo. The residue was dissolved in MeOH (10 mL) and CsF (2 eq.) in MeOH (5 mL) was added. The precipitate formed solid was filtered, washed with MeOH (2 × 30 mL) and air dried to give the desired products **9**–**11**.

### 3.4. Synthesis of [(H(CH_2_[COCH=CH(OCH_3_)C_6_H_3_O]_2_))-CH_2_-C-CH-N_3_((CH_2_)_2_O)_2_]-(B_12_H_11_)]Cs_2_ ***9***

Prepared from compound **4** (0.15 g, 0.20 mmol), alkynyl-curcumin **3** (0.08 g, 0.20 mmol), diisopropylethylamine (1 mL, 0.74 g, 5.73 mmol) and CuI (0.004 g, 0.02 mmol) in 20 mL of ethanol. The product was obtained as a a white solid of **9** (0.12 g, yield 67%). ^1^H-NMR (400 MHz, DMSO-*d*_6_): δ 8.38 (1H, s, -C*H*CN_3_), 7.50 (2H, m, 2×-C*H*=CH-), 7.26 (4H, m, 4×-C*H*=C *in phehyl*), 7.06 (2H, m, 2×-CH=C*H*-), 6.80 (1H, m, -C=C*H- in phehyl*), 6.63 (1H, m, C=C*H- in phehyl*), 6.00 (1H, br.s, =C*H*-C), 5.18 (2H, s, -OC*H_2_-*C), 4.56 (2H, s, OC*H_2_*-), 3.81 (3H, s, C*H_3_*OC_6_H_3_-), 3.78 (3H, s, C*H_3_*OC_6_H_3_-), 3.41 (6H, m, 2×-OC*H*_2_, C*H_2_*N_3_), 1.5–0.5 (br.m, BH) ppm; ^11^B-NMR (128 MHz, DMSO-*d*_6_): 6.3 (1B, s, B-O), −16.8 (5B, d, *J* = 144), −18.1 (5B, d, *J* = 139), −22.7 (1B, d, *J* = 124) ppm. ^13^C-NMR (101 MHz, DMSO-*d*_6_): 184.4 (-C=O), 183.0 (-C=O), 149.9 (=*C*-O-CH_3_ *in phehyl*), 149.8 (=*C*-O-CH_3_ *in phehyl*), 149.0 (=*C*-O-CH_2_- *in phehyl*), 148.5 (=*C*-OH *in phehyl*), 141.5 (-*C*H=CH-), 140.8 (-*C*H=CH-), 140.4 (*C*N_3_CH), 129.0 (=*C*H-C=O), 126.7 (=*C*H-C=O), 123.7 (=*C*-CH- *in phehyl*), 123.0 (-*C*=CH- *in phehyl*), 122.8 (=*C*H-CH- *in phehyl*), 122.0 (CN_3_*C*H), 121.6 (-*C*H=CH- *in phehyl*), 116.2 (=*C*H-C-O-CH_3_), 114.1 (-*C*H=C-O-CH_3_), 111.8 (-*C*H=C-OH), 111.3 (=*C*H-C-O-CH_2_-), 72.0 (O*C*H_2_-), 69.3 (*C*H_2_O-), 67.2 (O*C*H_2_-), 60.1 (O*C*H_2_-), 56.44 (O-CH_3_), 56.15 (O-CH_3_), 56.11 (*C*H_2_-C=O), 50.5 (*C*H_2_N) ppm. HRMS (ESI) *m/z* for [C_28_H_41_B_12_N_3_O_8_Cs]^-^ calcd 810.3160, found: 810.3132.

### 3.5. Synthesis of [(H(CH_2_[COCH=CH(OCH_3_)C_6_H_3_O]_2_))-CH_2_-C-CH-N_3_(CH_2_)_5_O]-(B_12_H_11_)]Cs_2_ ***10***

Prepared from compound **5** (0.15 g, 0.20 mmol), alkynyl-curcumin **3** (0.08 g, 0.20 mmol), diisopropylethylamine (1 mL, 0.74 g, 5.73 mmol) and CuI (0.004 g, 0.02 mmol) in 20 mL of ethanol. The product was obtained as a a white solid of **10** (0.12 g, yield 64%). ^1^H-NMR (400 MHz, DMSO) δ 8.27 (1H, s, -C*H*CN_3_), 7.52 (2H, m, 2×-C*H*=CH-), 7.22 (6H, m, 4×-C*H*=C *in phehyl*, 2×-CH=C*H*-), 6.77 (2H, m, 2×-C=C*H- in phehyl*), 6.06 (1H, br.s, =C*H*-C), 5.17 (2H, s, -OC*H_2_-*C), 4.34 (2H, s, BOC*H*_2_), 3.81 (6H, s, 2×C*H_3_*OC_6_H_3_-), 3.24 (2H, s, -C*H_2_*), 1.80 (2H, s, -C*H_2_*), 1.36 (2H, s, -C*H_2_*CH*_2_*N_3_), 1.19 (2H, s, -CH_2_C*H_2_*N_3_), 1.5–0.5 (br.m, BH) ppm; ^11^B-NMR (128 MHz, DMSO-*d*_6_): 6.5 (1B, s, B-O), −16.8 (5B, d, *J* = 131), −18.3 (5B, d, *J* = 130), −22.9 (1B, unsolved d) ppm. ^13^C-NMR (101 MHz, acetone-*d*_6_): 183.7 (-C=O), 182.9 (-C=O), 149.7 (=*C*-O-CH_3_ *in phehyl*), 149.1 (=*C*-O-CH_3_ *in phehyl*), 148.4 (=*C*-O-CH_2_- *in phehyl*), 147.9 (=*C*-OH *in phehyl*), 142.6 (-*C*H=CH-), 141.7 (-*C*H=CH-), 140.8 (*C*N_3_CH), 128.8 (=*C*H-C=O), 125.8 (=*C*H-C=O), 125.3 (=*C*-CH- *in phehyl*), 124.5 (-*C*=CH- *in phehyl*), 124.1 (=*C*H-CH- *in phehyl*), 122.9 (CN_3_*C*H), 120.9 (-*C*H=CH- *in phehyl*), 116.6 (=*C*H-C-O-CH_3_), 113.8 (-*C*H=C-O-CH_3_), 111.5 (-*C*H=C-OH), 111.1 (=*C*H-C-O-CH_2_-), 68.2 (O*C*H_2_-), 62.1 (*C*H_2_O-), 56.0 (O-*C*H_3_), 55.9 (O-*C*H_3_), 54.5 (*C*H_2_-C=O), 50.0 (N*C*H_2_-), 31.6 (*C*H_2_-), 30.3 (*C*H_2_-), 23.3 (*C*H_2_-) ppm. HRMS (ESI) *m/z* for [C_29_H_43_B_12_N_3_O_7_Cs]^-^ calcd 808.3368, found: 808.3347.

### 3.6. Synthesis of [(H(CH_2_[COCH=CH(OCH_3_)C_6_H_3_O]_2_))-CH_2_-C-CH-N_3_(CH_2_)_4_O]-(B_12_H_11_)]Cs_2_ ***11***

Prepared from compound **5** (0.20 g, 0.27 mmol), alkynyl-curcumin **3** (0.11 g, 0.27 mmol), diisopropylethylamine (1 mL, 0.74 g, 5.73 mmol) and CuI (0.004 g, 0.02 mmol) in 20 mL of ethanol. The product was obtained as a a white solid of **10** (0.16 g, yield 62%). ^1^H-NMR (400 MHz, DMSO) δ 8.26 (s, 1H, -C*H*CN_3_), 7.55 (2H, m, 2×-C*H*=CH-), 7.34 (2H, d, 2×-C*H*=C *in phehyl*), 7.23 (4H, m, 2×-C*H*=C *in phehyl*, 2×-CH=C*H*-), 7.10 (1H, m, C=C*H- in phehyl*), 6.77 (1H, m, C=C*H- in phehyl*), 6.06 (1H, br.s, =C*H*-C), 5.17 (s, 2H, -OC*H_2_-*C), 4.40 (2H, t, -BOC*H*_2_, *J* = 5.4 Hz), 3.82 (6H, s, 2×C*H_3_*OC_6_H_3_-), 3.32 (2H, s, -OCH_2_C*H*_2_-), 1.78 (2H, s, -C*H_2_*CH*_2_*N_3_), 1.30 (2H, s, -CH_2_C*H_2_*N_3_), 1.5–0.5 (br.m, BH) ppm; ^11^B-NMR (128 MHz, DMSO-*d*_6_): 6.4 (1B, s, B-O), −16.8 (5B, d, *J* = 140), −18.3 (5B, d, *J* = 146), −22.8 (1B, unsolved d) ppm. ^13^C-NMR (101 MHz, acetone-*d*_6_): 183.5 (-C=O), 182.6 (-C=O), 149.9 (=*C*-O-CH_3_ *in phehyl*), 149.7 (=*C*-O-CH_3_ *in phehyl*), 148.8 (=*C*-O-CH_2_- *in phehyl*), 148.4 (=*C*-OH *in phehyl*), 142.4 (-*C*H=CH-), 141.5 (-*C*H=CH-), 140.2 (*C*N_3_CH), 128.6 (=*C*H-C=O), 125.8 (=*C*H-C=O), 125.5 (=*C*-CH- *in phehyl*), 124.1 (-*C*=CH- *in phehyl*), 123.1 (=*C*H-CH- *in phehyl*), 122.0 (CN_3_*C*H), 120.9 (-*C*H=CH- *in phehyl*), 116.4 (=*C*H-C-O-CH_3_), 113.8 (-*C*H=C-O-CH_3_), 111.6 (-*C*H=C-OH), 111.1 (=*C*H-C-O-CH_2_-), 67.8 (O*C*H_2_-), 62.2 (*C*H_2_O-), 56.4 (O-*C*H_3_), 56.1 (O-*C*H_3_), 55.9 (*C*H_2_-C=O), 50.0 (N*C*H_2_-), 28.8 (*C*H_2_-), 28.1 (*C*H_2_-) ppm. HRMS (ESI) *m/z* for [C_28_H_41_B_12_N_3_O_7_Cs]^-^ calcd 794.3211, found: 794.3205.

### 3.7. Biological Studies

The assessment of antimicrobial activity according to the values of the minimum inhibitory concentration (MIC) was carried out in relation to activity against Gram-negative microorganisms (including reference strains and clinical isolates of *Acinetobacter baumannii* strain 73 resistant to beta-lactam antibiotics), Gram-positive microorganisms (including methicillin-resistant clinical isolate of *Staphylococcus aureus* strain 17), fungi of the genus *Candida* (clinical isolates resistant to fluconazole) and reference strain *Aspergillus fumigatus* ATCC 46645. All strains were obtained from the Medical Microbiology Laboratory of State Research Center for Antibiotics (Moscow, Russia). For MIC analysis, the broth microdilution method in a 96-well plate was used based on standard recommendations [62,63,64].

All test compounds were dissolved in dimethyl sulfoxide (DMSO) in the volume necessary to obtain a concentration of 100,000 μg/mL. They were further diluted to a concentration of 2000 μg/mL in Mueller–Hinton nutrient broth for analysis of bacterial test cultures and RPMI1640 medium with l-glutamine containing 2% glucose for analysis of fungal cultures. A series of two-fold dilutions of the test samples was prepared in 96-well plates in a volume of 50 μL for assessing the activity of bacterial cultures and 100 μL for fungal cultures. The range of working concentrations was 1000–0.48 mg/L. The concentration of DMSO in the dilution of drugs 1000 µ/mL–1%. MIC values were determined visually after the appropriate incubation time for the test organisms at 36 ± 1 °C compared to a growth control without samples.

## 4. Conclusions

Five novel anionic conjugates of cobalt bis(dicarbollide) and *closo*-dodecaborate with curcumin were synthesized by the copper(I)-catalyzed azide-alkyne cycloaddition. The resulting conjugates differed in antimicrobial activity from the curcumin base compound. All derivatives were active when exposed to *Bacillus cereus* ATCC 10702 and were not active against Gram-negative microorganisms and *Candida albicans* at the maximum studied concentration of 1000 mg/L. Only derivative **5** exhibited activity that is 2–4 times superior to curcumin against Gram-positive microorganisms: *Staphylococcus aureus* ATCC 29213, *Enterococcus faecalis* ATCC 29212 and the clinical isolate MRSA 17. Unlike curcumin, this derivative was also active against *Aspergillus fumigatus* ATCC 46645. Thus, the obtained results show the potential use of cobalt bis(dicarbollide) and *closo*-dodecaborate conjugates based on curcumin as antibacterial agents.

## Figures and Tables

**Table 1:** In vitro comparative assessment of the antimicrobial activities of curcumin and its boron clusters conjugates.

Organism	MIC (mg/L)					
	Curcumin	4	5	9	10	11
Gram-negative bacteria						
*Escherichia coli* ATCC 25922	>1000	>1000	>1000	>1000	>1000	>1000
*Pseudomonas aeruginosa* ATCC 27853	>1000	>1000	>1000	>1000	>1000	>1000
*Acinetobacter baumannii* 73	>1000	>1000	>1000	>1000	>1000	>1000
Gram-positive bacteria						
*Staphylococcus aureus* ATCC 29213	62.5	1000	31.25	>1000	>1000	>1000
*Staphylococcus aureus* (MRSA) 17	125	1000	31.25	>1000	>1000	>1000
*Bacillus cereus* ATCC 10702	125	500	62.5	500	250	250
*Enterococcus faecalis* ATCC 29212	>1000	>1000	250	>1000	>1000	>1000
Fungal streins						
*Candida albicans* 604M	>1000	>1000	>100	>1000	>1000	>1000
*Candida albicans* 8P	>1000	>1000	>1000	>1000	>1000	>1000
*Aspergillus fumigatus* ATCC 46645	>1000	>1000	125	>1000	>1000	>1000

## Data Availability

The data presented in this study are available in Supplementary Materials.

## Supplementary Materials

The following supporting information can be downloaded at: https://www.mdpi.com/article/10.3390/molecules27092920/s1, Figures S1–S5: ESI-HRMS spectra of compounds **4**, **5** and **9**–**11**, Figures S6-–S10: IR spectra of compounds **4**, **5** and **9**–**11**, Figures S11–S18: ^1^H, ^11^B{^1^H}, ^11^B and ^13^C spectra of compounds **4** and **5** in acetone-*d*_6_, Figures S19–S30: ^1^H, ^11^B{^1^H}, ^11^B and ^13^C spectra of compounds **9**–**11** in DMSO-*d*_6_.

## Sample Availability

Samples of the compounds are available from the authors.
