# Supplementary material for: Synthesis and Antibacterial Activity Studies of the Conjugates of Curcumin with closo-Dodecaborate and Cobalt Bis(Dicarbollide) Boron Clusters
Source: Molecules. 2022 May 3;27(9):2920. doi: 10.3390/molecules27092920 (PMC9101702; doi:10.3390/molecules27092920)
Supplement: Supplementary file 1 [file molecules-27-02920-s001.zip › molecules-1680436-supplementary.pdf]

SUPPORTING INFORMATION

Synthesis and Antibacterial Activity Studies of the Conjugates of Curcumin with *closo*-Dodecaborate and Cobalt Bis(Dicarbollide) Boron Clusters §

Anna A. Druzina [1,*], Natalia E. Grammatikova [2], Olga B. Zhidkova [1], Anastasia A. Nekrasova [1,3], Nadezhda V. Dudarova [1], Irina D. Kosenko [1], Mikhail A. Grin [3], Vladimir I. Bregadze [1]

1 A.N. Nesmeyanov Institute of Organoelement Compounds, Russian Academy of Sciences, 28 Vavilov Str., 119991 Moscow, Russia; nekrasova_na@list.ru (N.A.N); nadezjdino_96@mail.ru (N.V.D.); Zolga57@mail.ru (O.B.Z.); kosenko@ineos.ac.ru (I.D.K.); sivaev@ineos.ac.ru (I.B.S.); bre@ineos.ac.ru (V.I.B)

2 Gause Institute of New Antibiotics, 11 B. Pirogovskaya, 119021, Moscow, Russia; ngrammatikova@yandex.ru (NEG)

3 M.V. Lomonosov Institute of Fine Chemical Technology, MIREA—Russian Technological University, 86 Vernadsky Av., 119571 Moscow, Russia; michael_grin@mail.ru (M.A.G)

* Correspondence: ilinova_anna@mail.ru (A.A.D.); Tel.: +7-926-404-5566

§ Dedicated to Professor Valery Petrosyan on the occasion of his 80th Jubilee and in recognition of his outstanding contributions to physical organic and organometallic chemistry.

^{1}H, ^{11}B and ^{13}C NMR, IR and high-resolution mass spectra of compounds **4, 5, 9-11**

Display Report

Analysis Info

Analysis Name	D:\Data\Chizhov\INEOS\Bregadze\Laskova\Nov_01_2021_1\da-039_&clb-.d	Acquisition Date	01.11.2021 13:50:37
Method	tune_wide_neg.m	Operator	BDAL@DE
Sample Name	/CHIZ DA-039	Instrument / Ser#	micrOTOF 10248
Comment	CH3OH 100 %, dil. 2000, calibrant added		

Acquisition Parameter

Source Type	ESI	Ion Polarity	Negative	Set Nebulizer	0.4 Bar
Focus	Not active			Set Dry Heater	180 °C
Scan Begin	50 m/z	Set Capillary	3200 V	Set Dry Gas	4.0 l/min
Scan End	3000 m/z	Set End Plate Offset	-500 V	Set Divert Valve	Waste

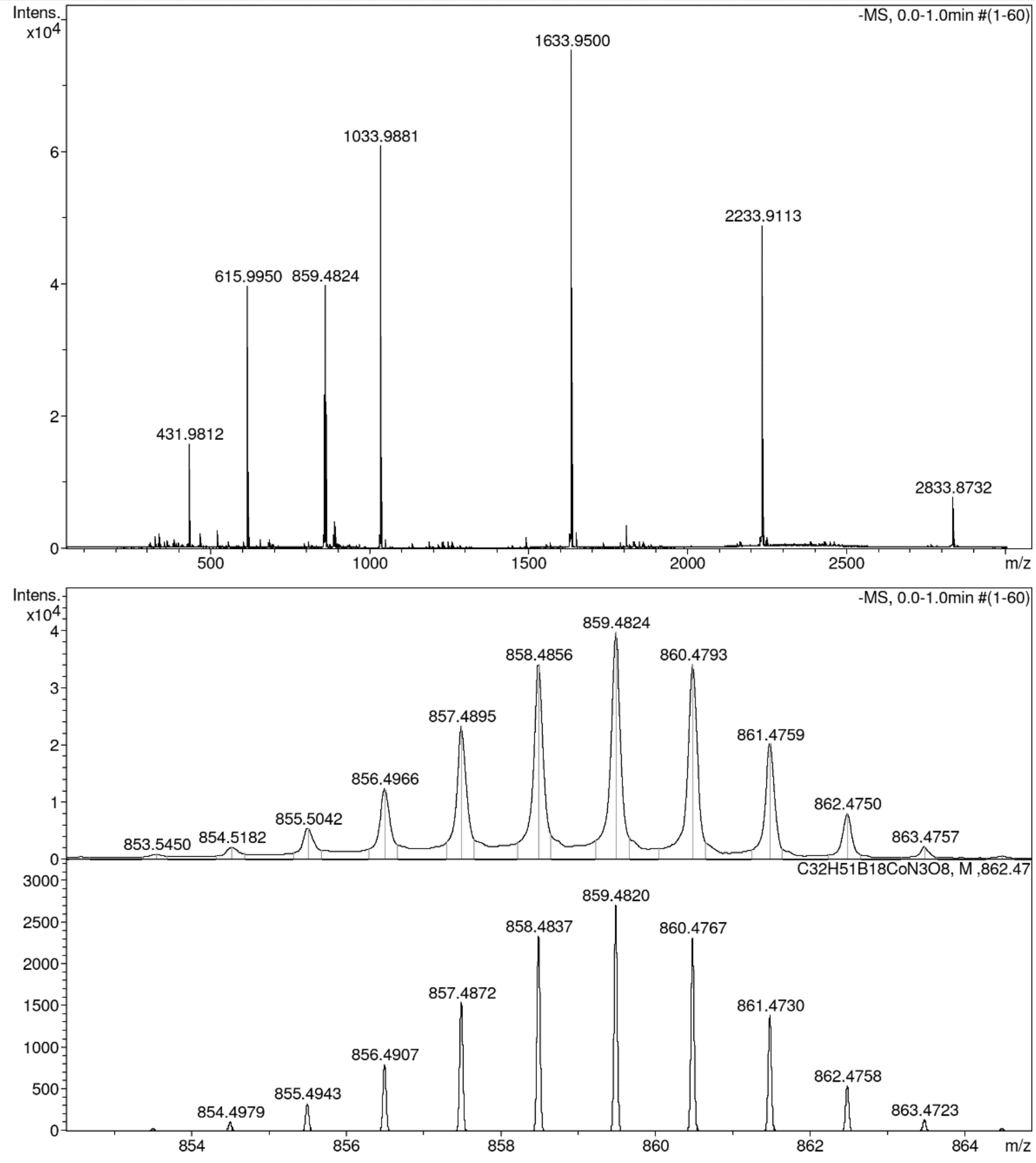

Bruker Compass DataAnalysis 4.0 printed: 01.11.2021 14:28:10 Page 1 of 1

Figure S1. ESI-HRMS spectrum of compound **4**

Display Report

Analysis Info

Analysis Name	D:\Data\Chizhov\INEOS\Bregadze\Druzina\Jan_17_2022\da-047_&clb-.d	Acquisition Date	17.01.2022 18:56:24
Method	tune_wide_neg.m	Operator	BDAL@DE
Sample Name	/CHIZ DA-047	Instrument / Ser#	micrOTOF 10248
Comment	CH3CN 100 %, dil. 200, calibrant added		

Acquisition Parameter

Source Type	ESI	Ion Polarity	Negative	Set Nebulizer	0.4 Bar
Focus	Not active			Set Dry Heater	180 °C
Scan Begin	50 m/z	Set Capillary	3200 V	Set Dry Gas	4.0 l/min
Scan End	3000 m/z	Set End Plate Offset	-500 V	Set Divert Valve	Waste

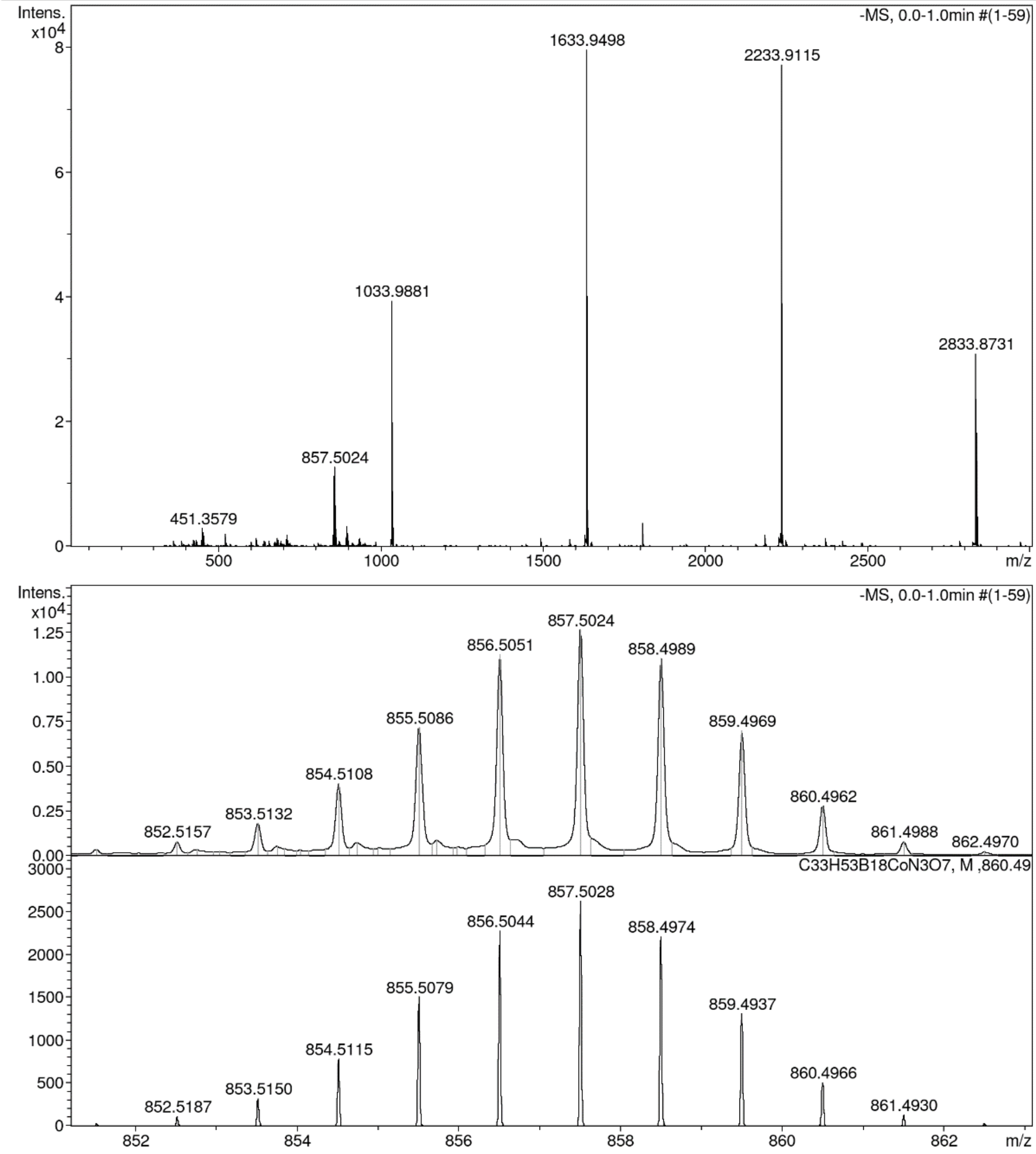

Bruker Compass DataAnalysis 4.0 printed: 17.01.2022 19:43:36 Page 1 of 1

Figure S2. ESI-HRMS spectrum of compound **5**

Display Report

Analysis Info

Analysis Name	C:\AOC2022\Druzina\Jan_17\da-048_&clb-.d	Acquisition Date	17.01.2022 19:06:37
Method	tune_wide_neg.m	Operator	BDAL@DE
Sample Name	/CHIZ DA-048	Instrument / Ser#	micrOTOF 10248
Comment	CH3CN 100 %, dil. 20, calibrant added		

Acquisition Parameter

Source Type	ESI	Ion Polarity	Negative	Set Nebulizer	0.4 Bar
Focus	Not active			Set Dry Heater	180 °C
Scan Begin	50 m/z	Set Capillary	3200 V	Set Dry Gas	4.0 l/min
Scan End	3000 m/z	Set End Plate Offset	-500 V	Set Divert Valve	Waste

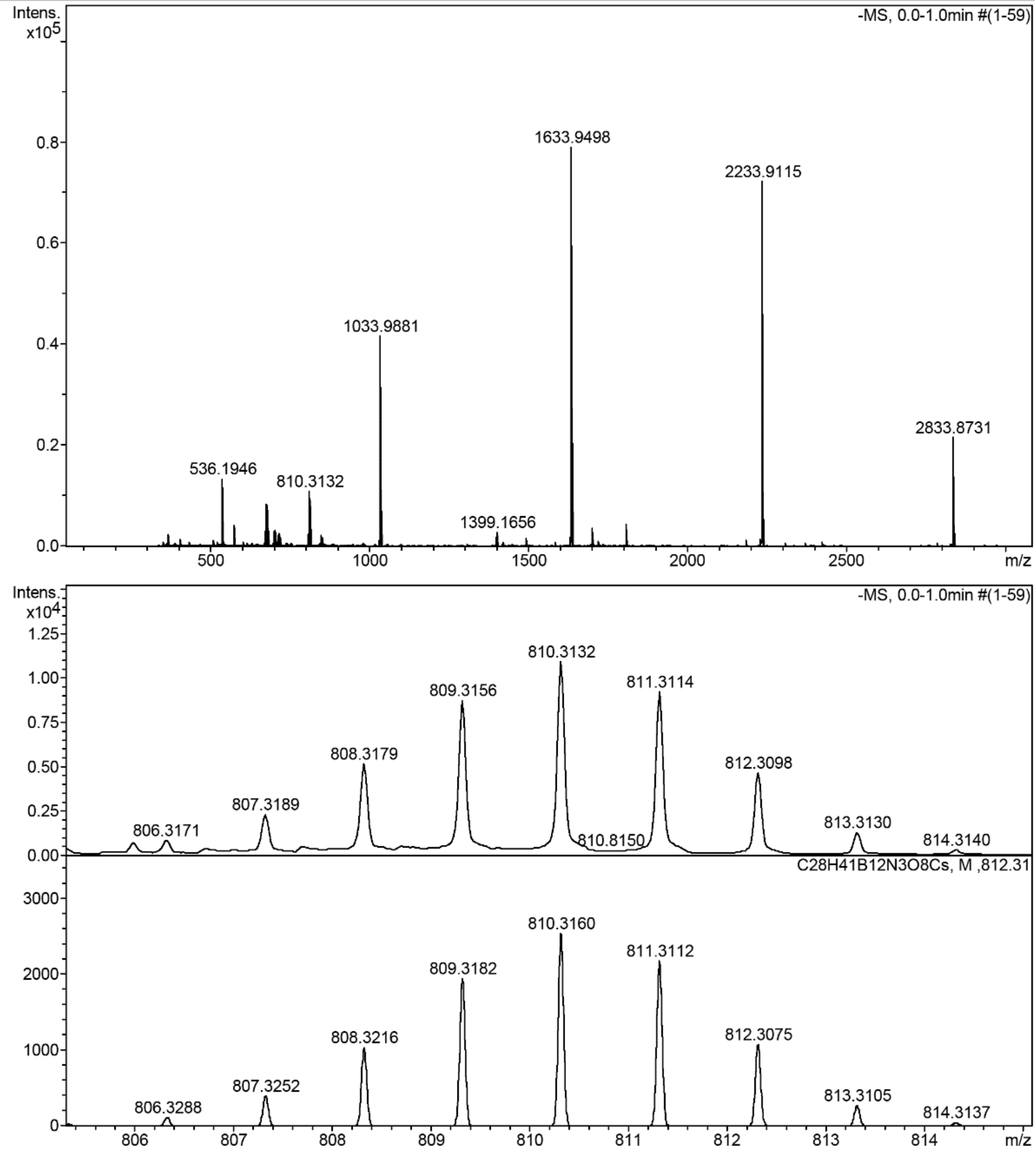

Bruker Compass DataAnalysis 4.0 printed: 18.01.2022 15:39:51 Page 1 of 1

Figure S3. ESI-HRMS spectrum of compound **9**

Display Report

Analysis Info

Analysis Name	C:\AOC2022\Druzina\Jan_17\da-050_&clb-.d	Acquisition Date	17.01.2022 19:17:16
Method	tune_wide_neg.m	Operator	BDAL@DE
Sample Name	/CHIZ DA-050	Instrument / Ser#	micrOTOF 10248
Comment	CH3CN 100 %, dil. 20, calibrant added		

Acquisition Parameter

Source Type	ESI	Ion Polarity	Negative	Set Nebulizer	0.4 Bar
Focus	Not active			Set Dry Heater	180 °C
Scan Begin	50 m/z	Set Capillary	3200 V	Set Dry Gas	4.0 l/min
Scan End	3000 m/z	Set End Plate Offset	-500 V	Set Divert Valve	Waste

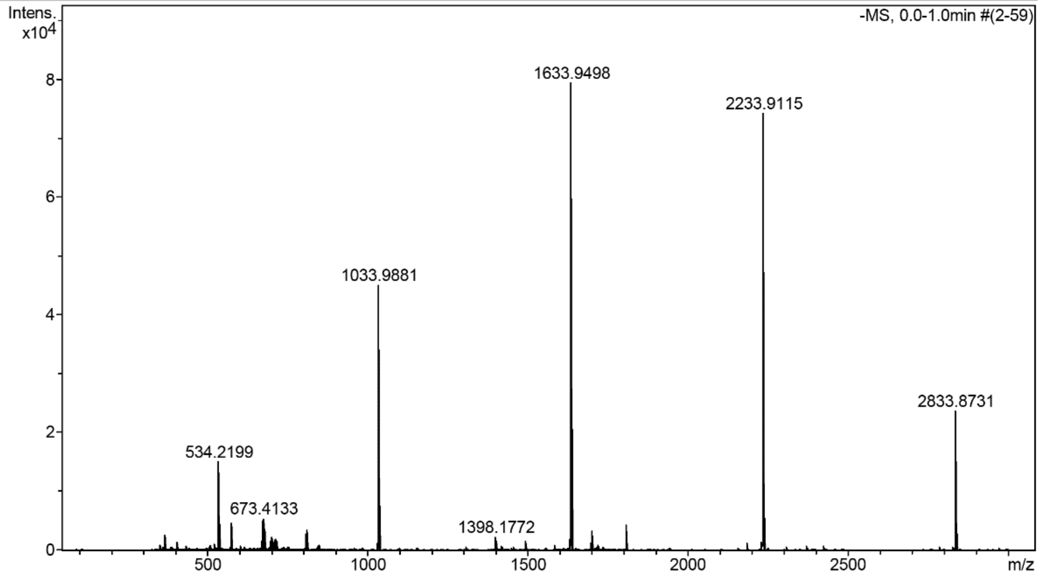

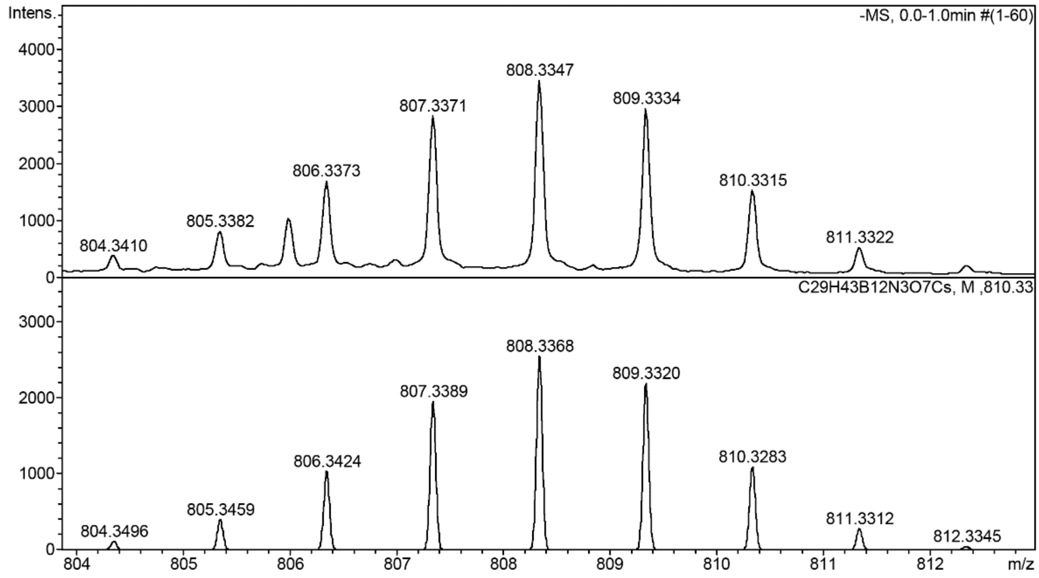

Bruker Compass DataAnalysis 4.0 printed: 18.01.2022 15:43:44 Page 1 of 1

Figure S4. ESI-HRMS spectrum of compound **10**

Display Report

Analysis Info

Analysis Name	C:\AOC2022\Druzina\Jan_17\da-049_&clb-.d	Acquisition Date	17.01.2022 19:12:24
Method	tune_wide_neg.m	Operator	BDAL@DE
Sample Name	/CHIZ DA-049	Instrument / Ser#	micrOTOF 10248
Comment	CH3CN 100 %, dil. 20, calibrant added		

Acquisition Parameter

Source Type	ESI	Ion Polarity	Negative	Set Nebulizer	0.4 Bar
Focus	Not active			Set Dry Heater	180 °C
Scan Begin	50 m/z	Set Capillary	3200 V	Set Dry Gas	4.0 l/min
Scan End	3000 m/z	Set End Plate Offset	-500 V	Set Divert Valve	Waste

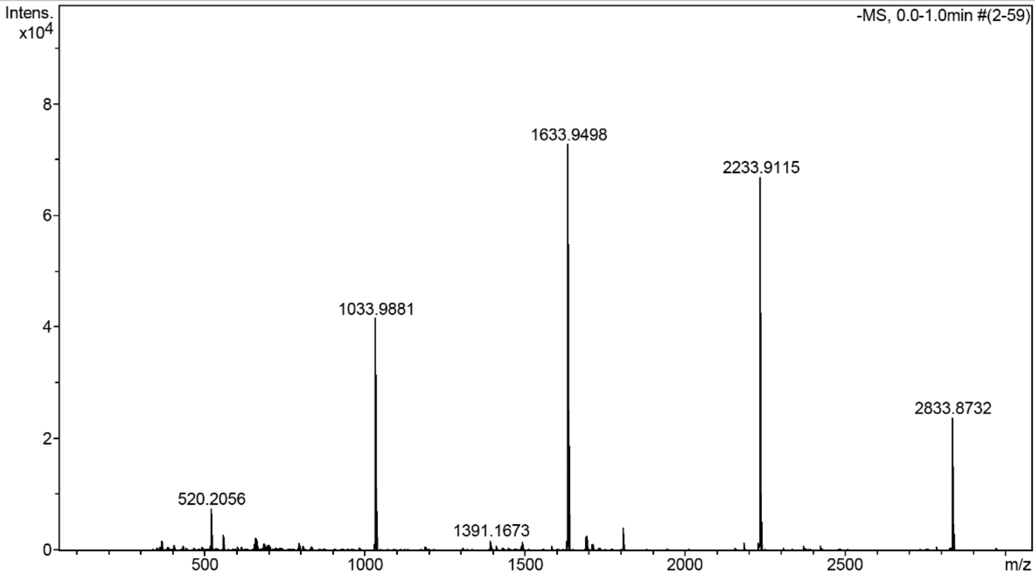

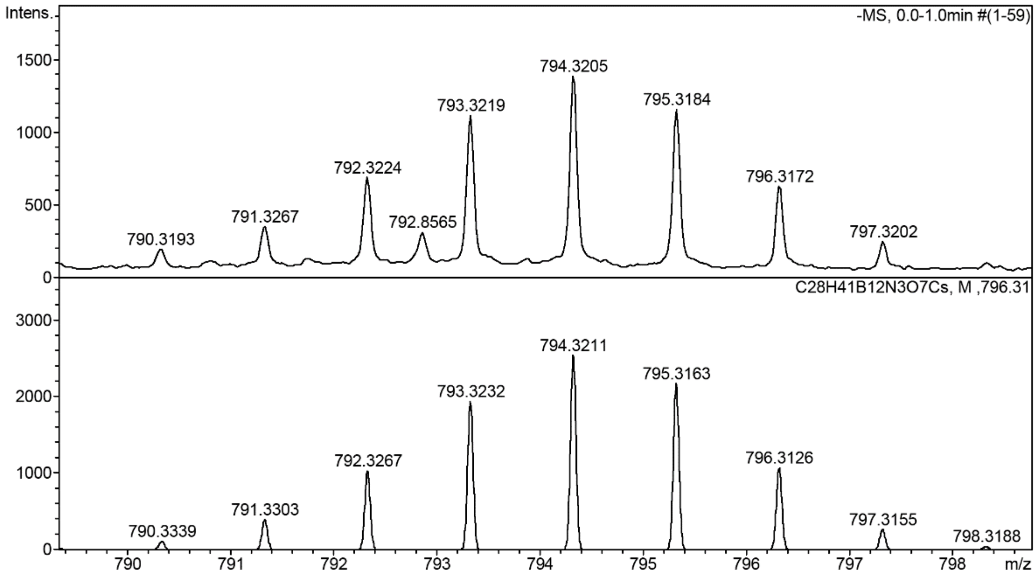

Figure S5. ESI-HRMS spectrum of compound **11**

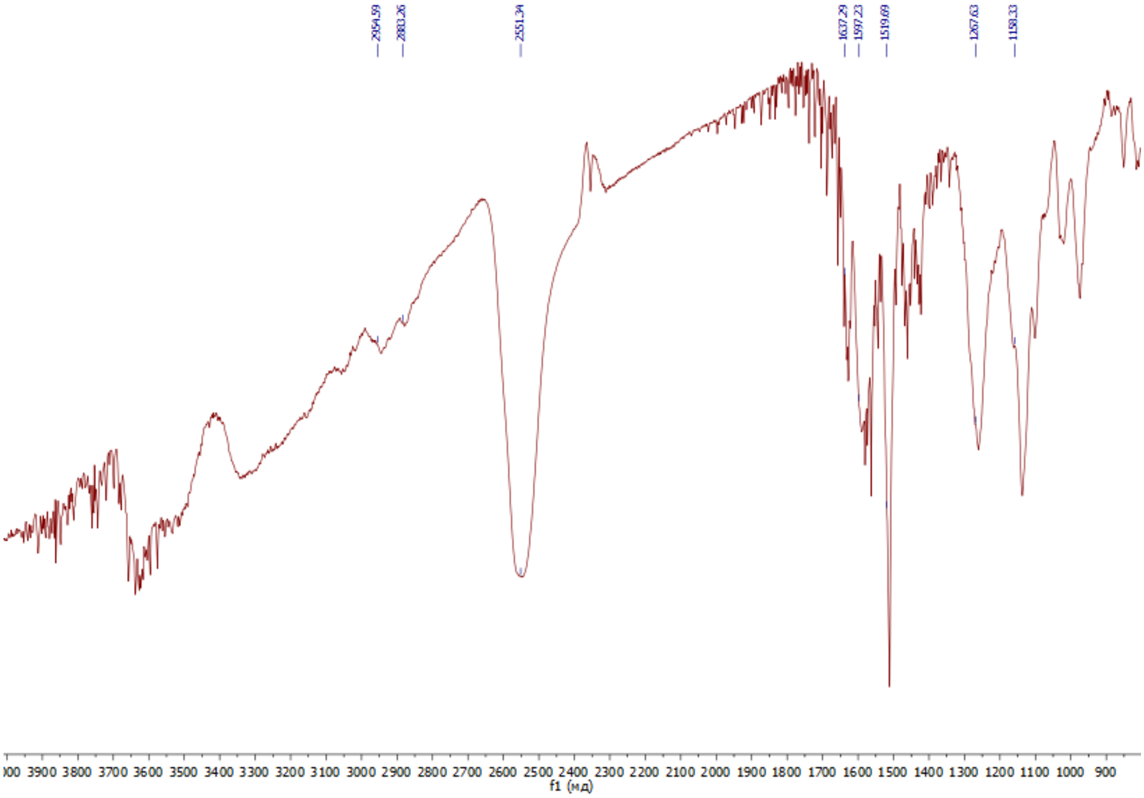

Figure S6. IR spectrum of compound **4**

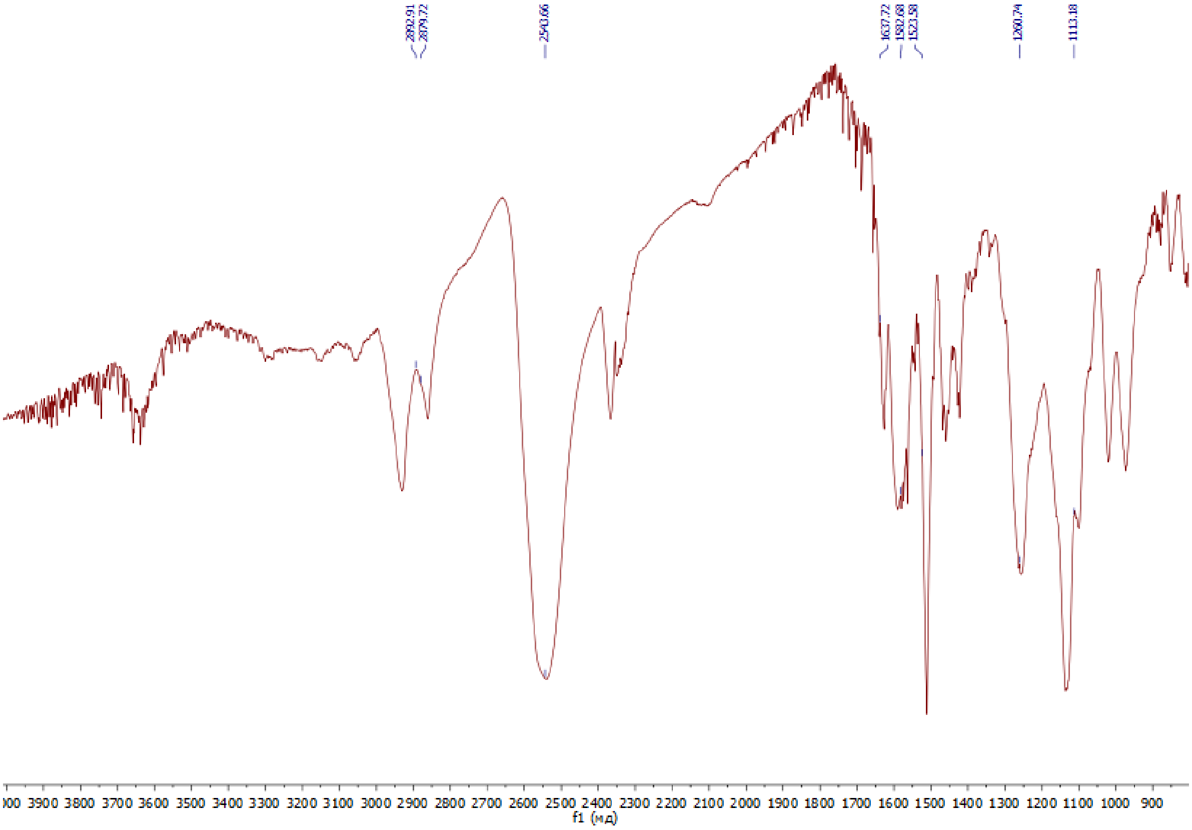

Figure S7. IR spectrum of compound **5**

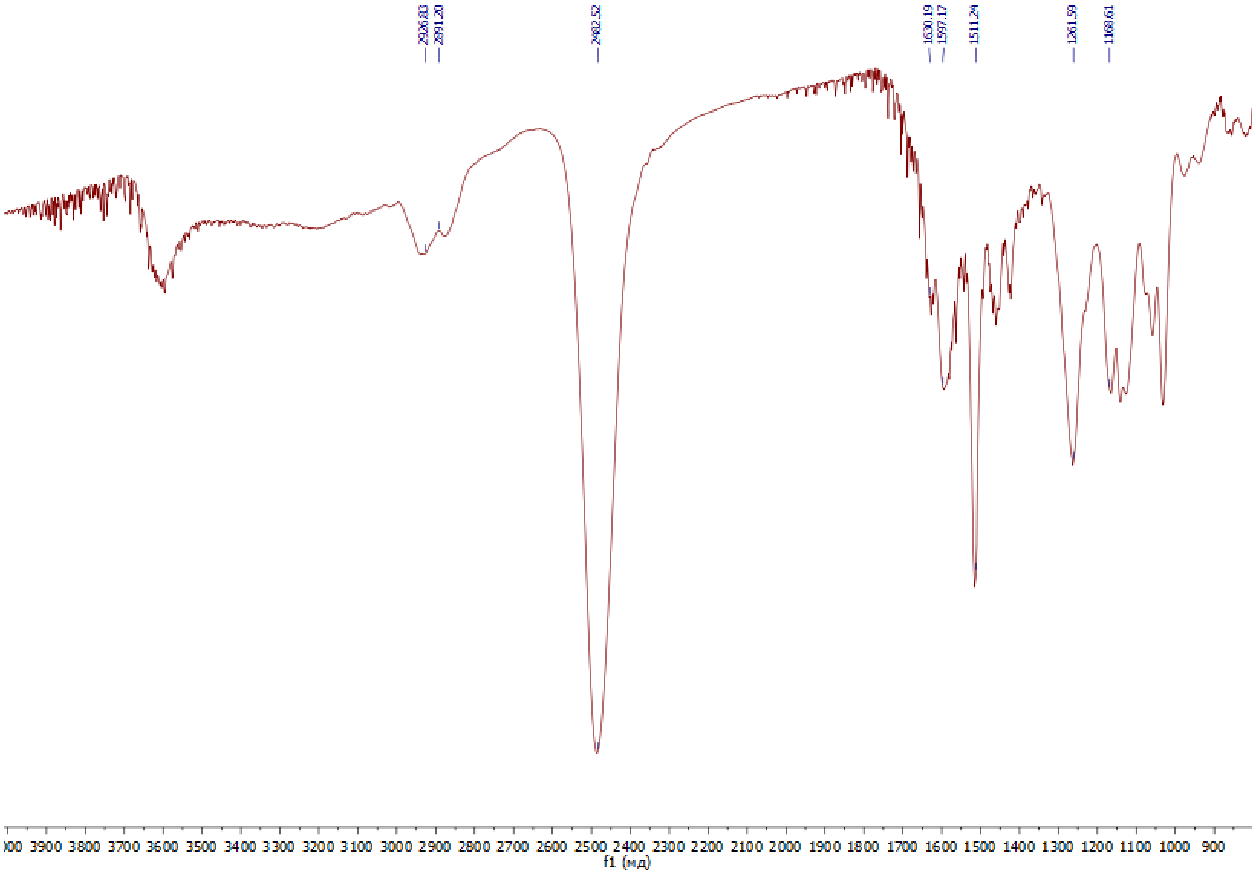

Figure S8. IR spectrum of compound **9**

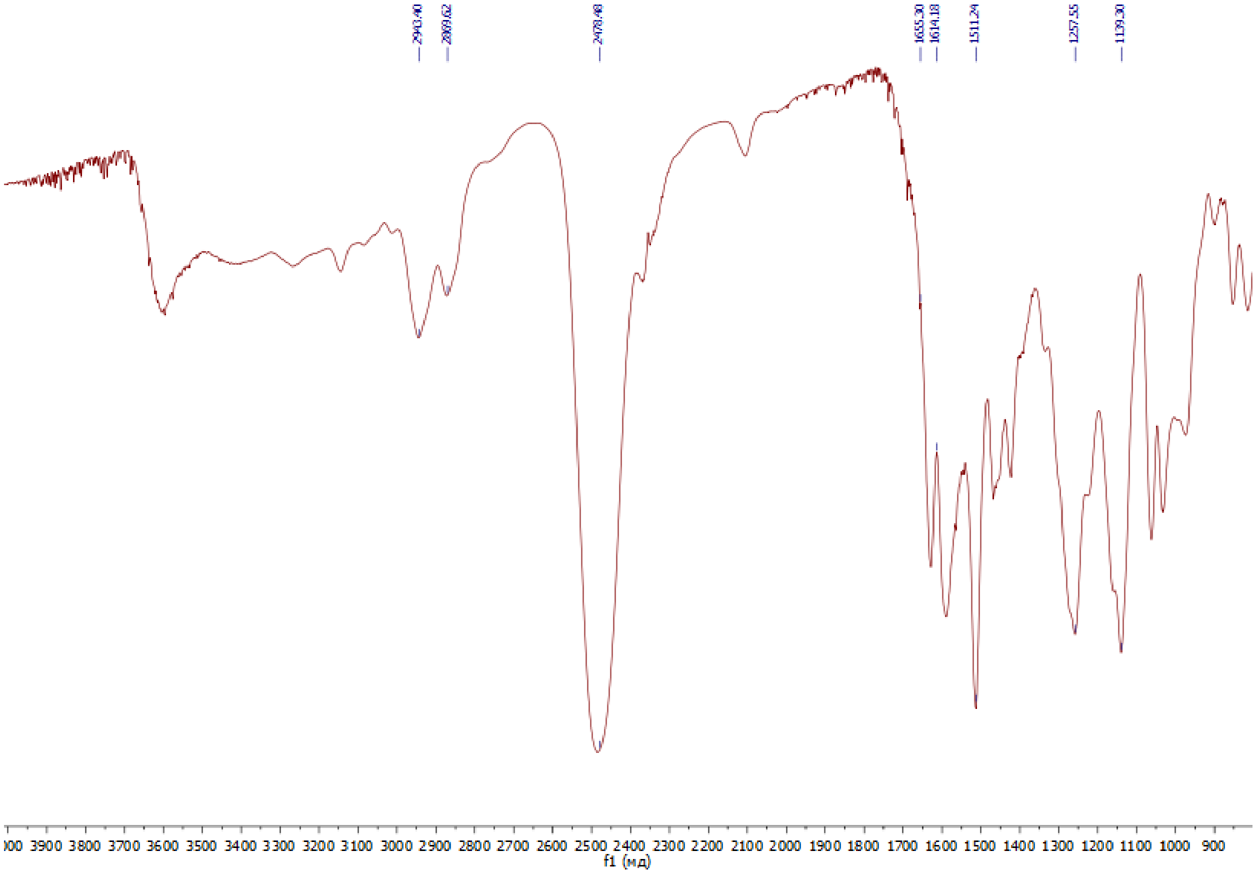

Figure S9. IR spectrum of compound **10**

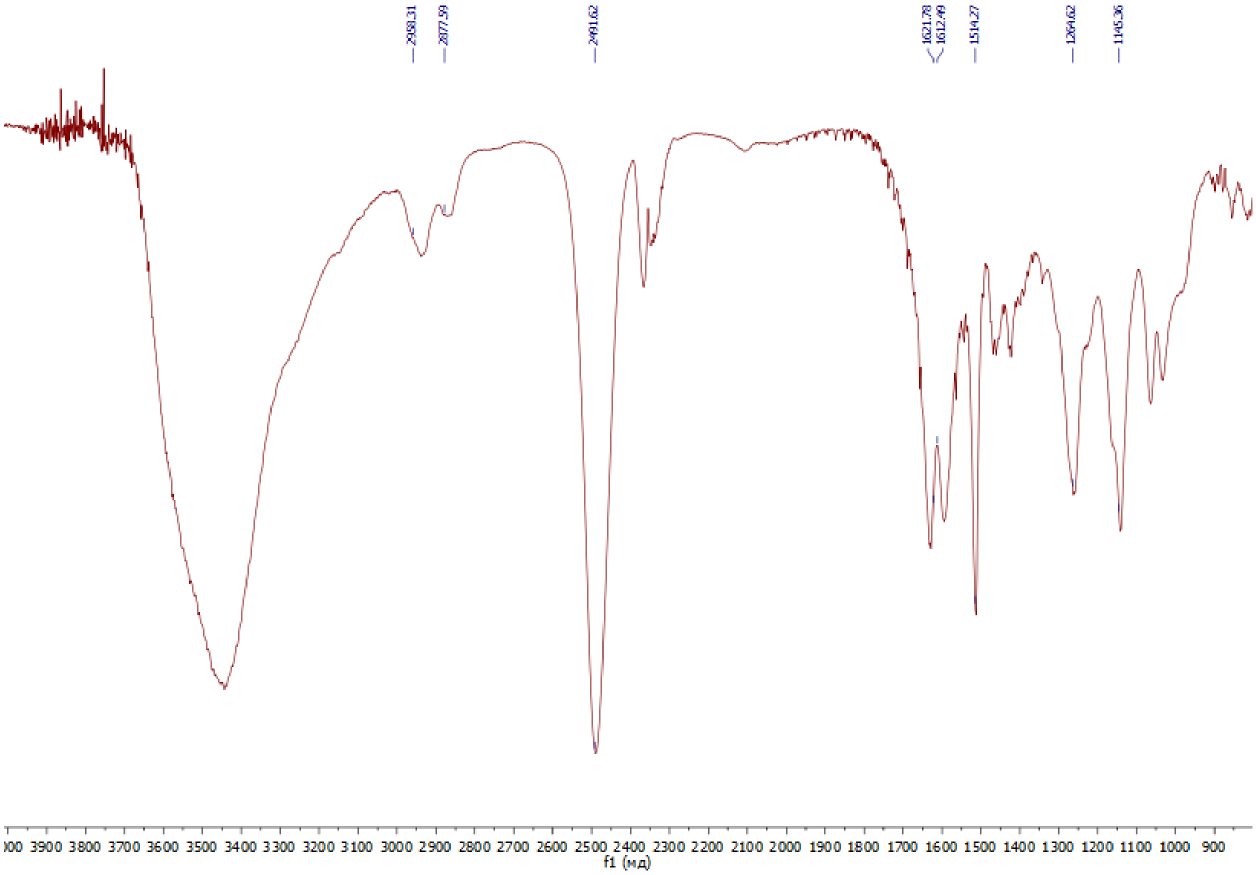

Figure S10. IR spectrum of compound **11**

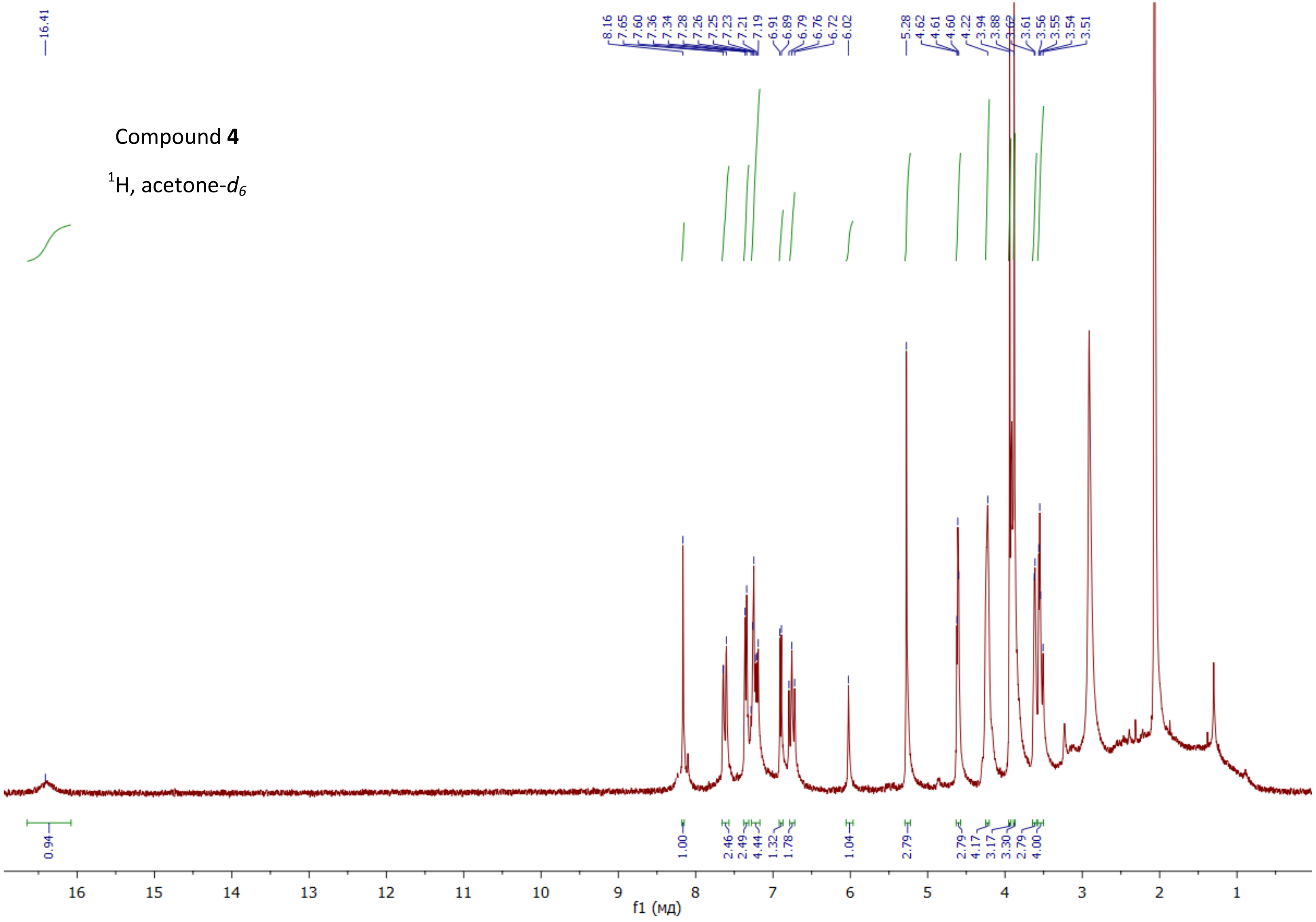

Figure S11. ^{1}H NMR spectrum of compound **4**

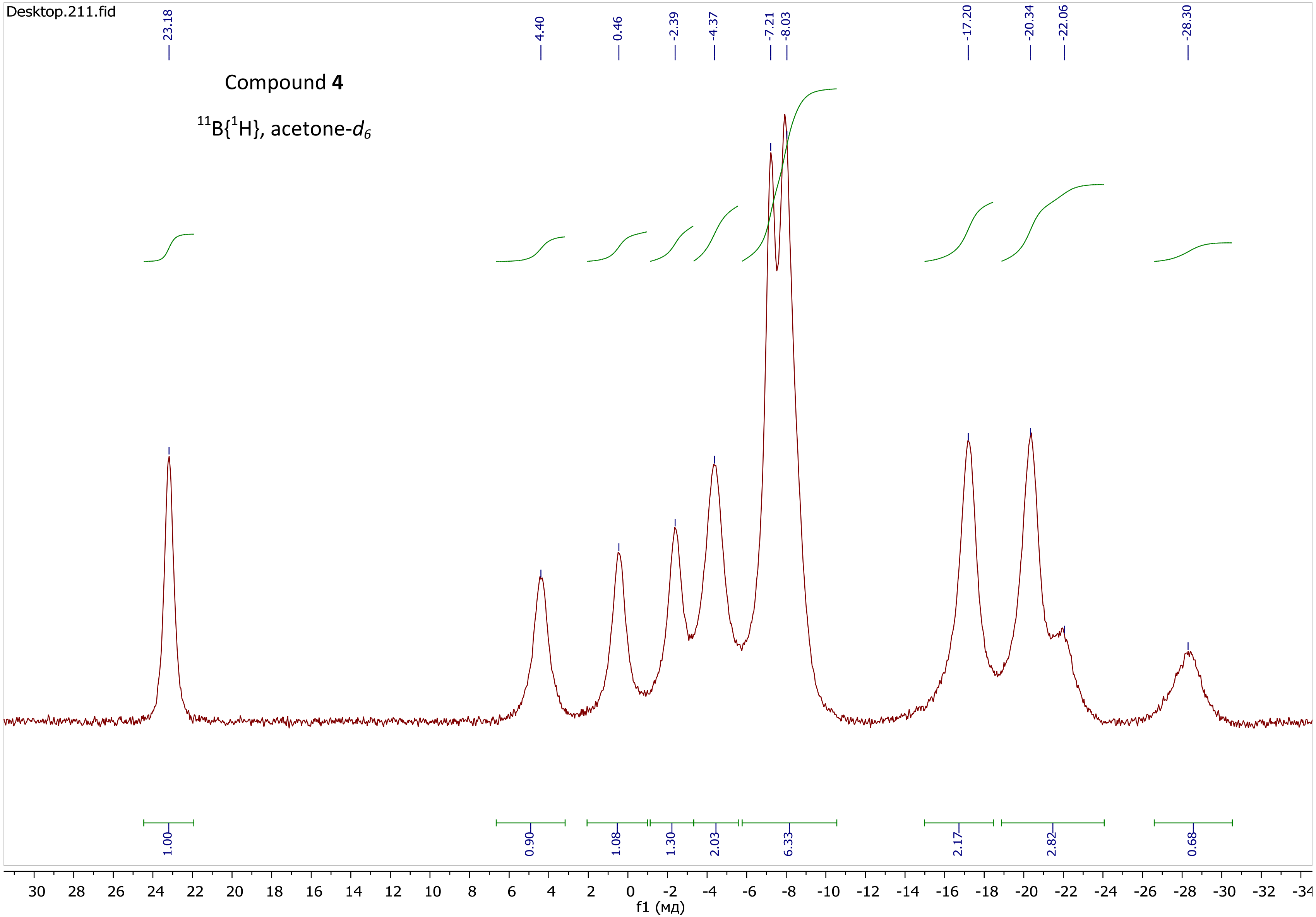

Figure S12. $^{11}B\{^{1}H\}$ NMR spectrum of compound **4**

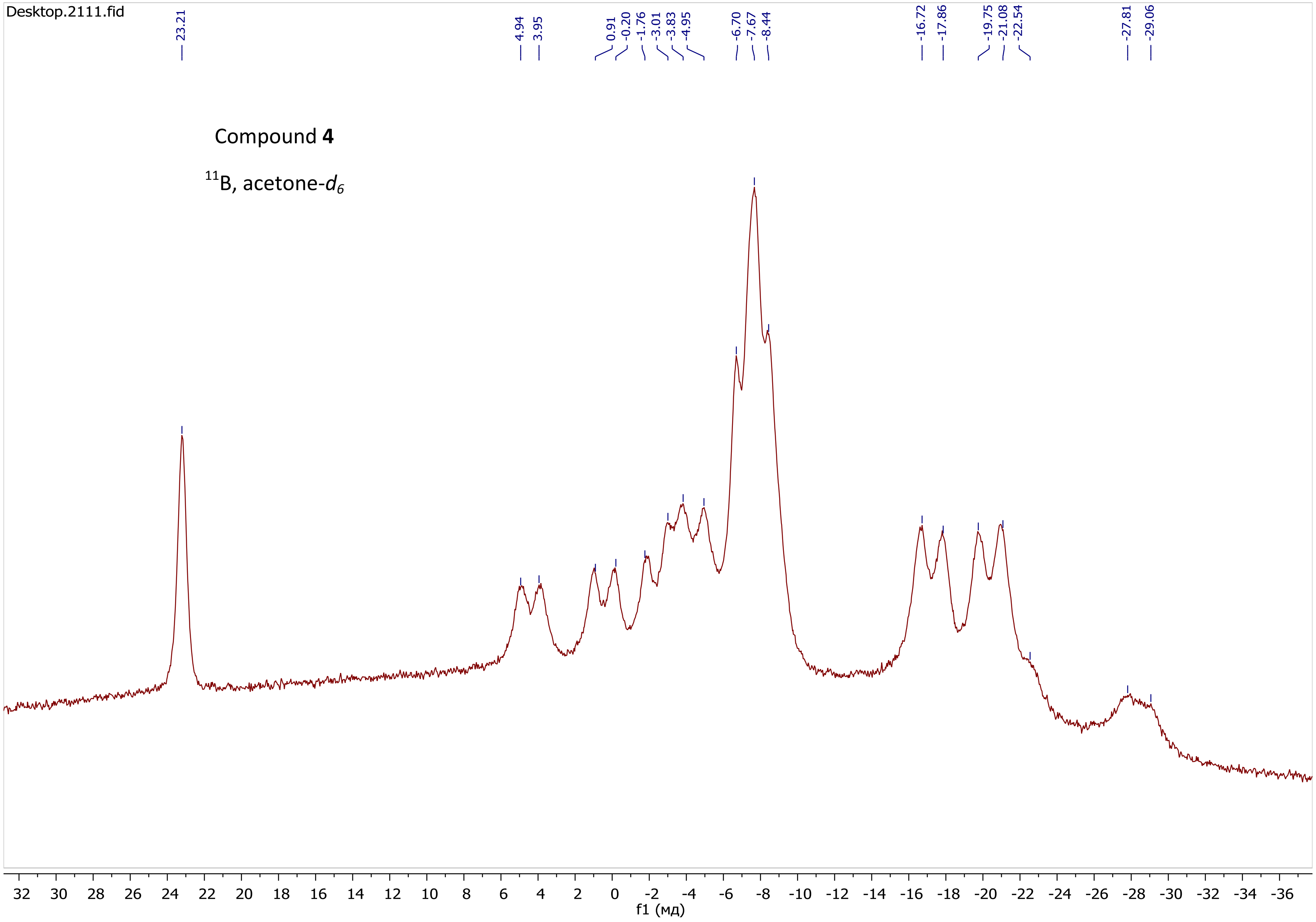

Figure S13. ^{11}B NMR spectrum of compound **4**

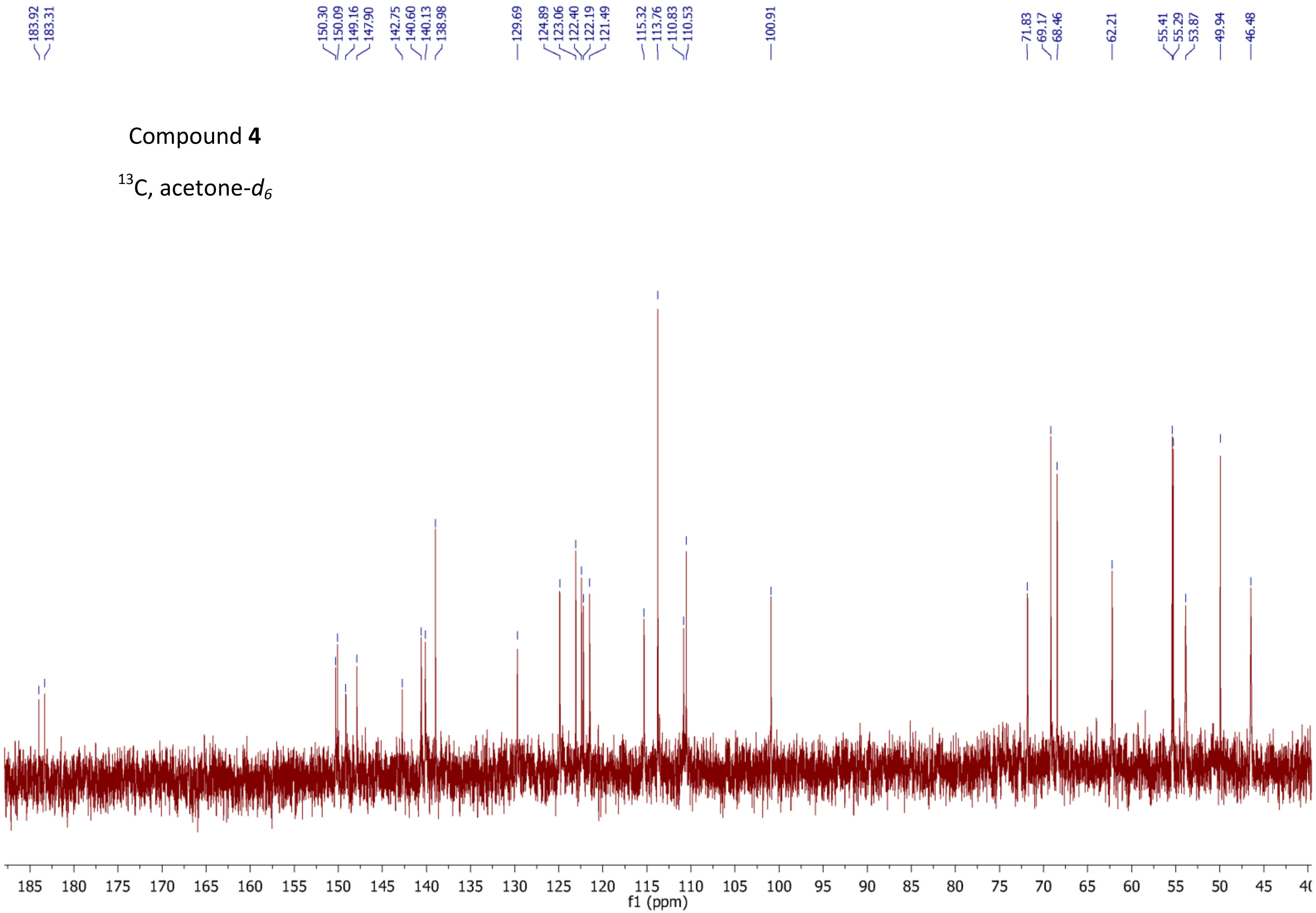

Figure S14. ^{13}C NMR spectrum of compound **4**

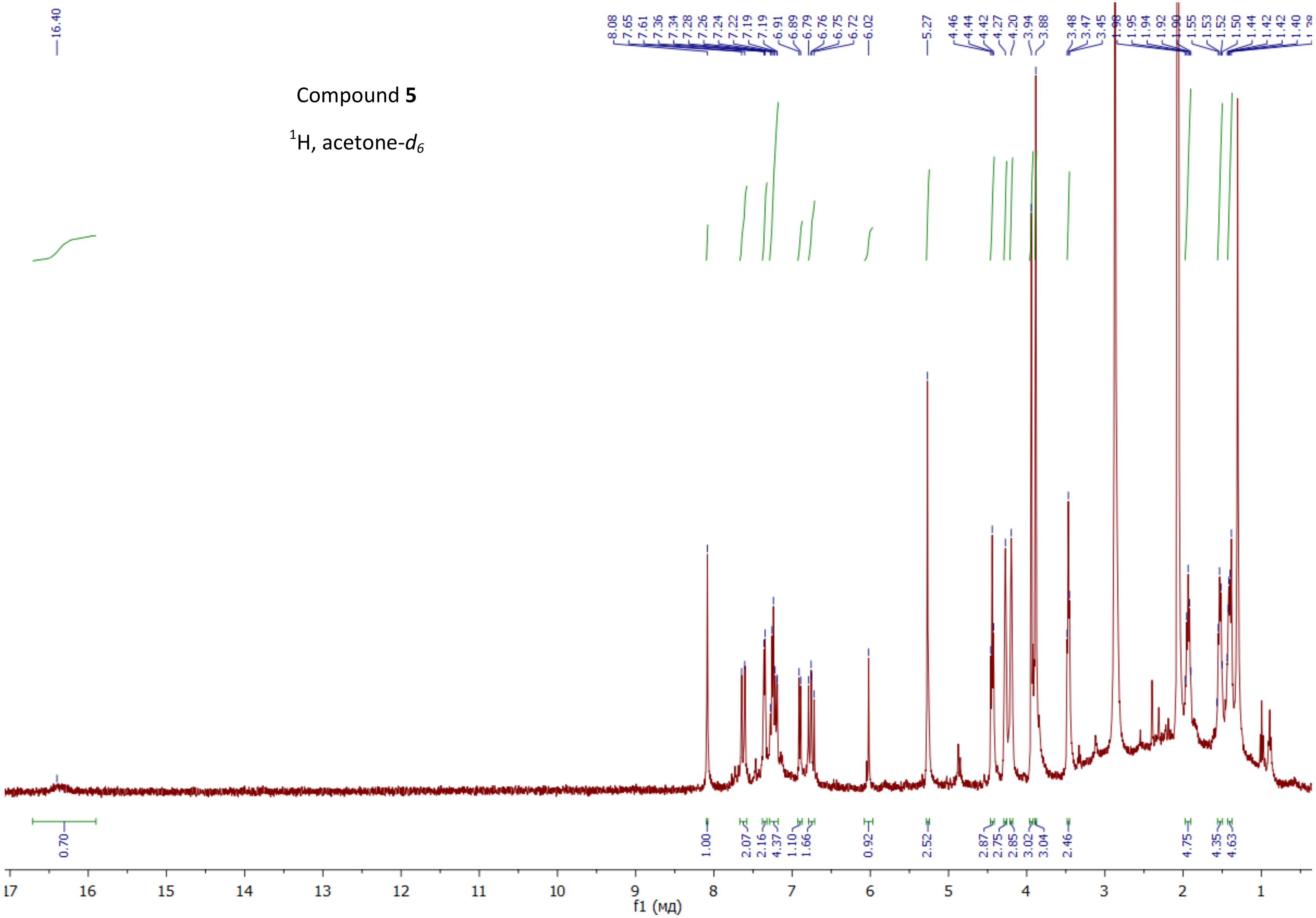

Figure S15. ^{1}H NMR spectrum of compound **5**

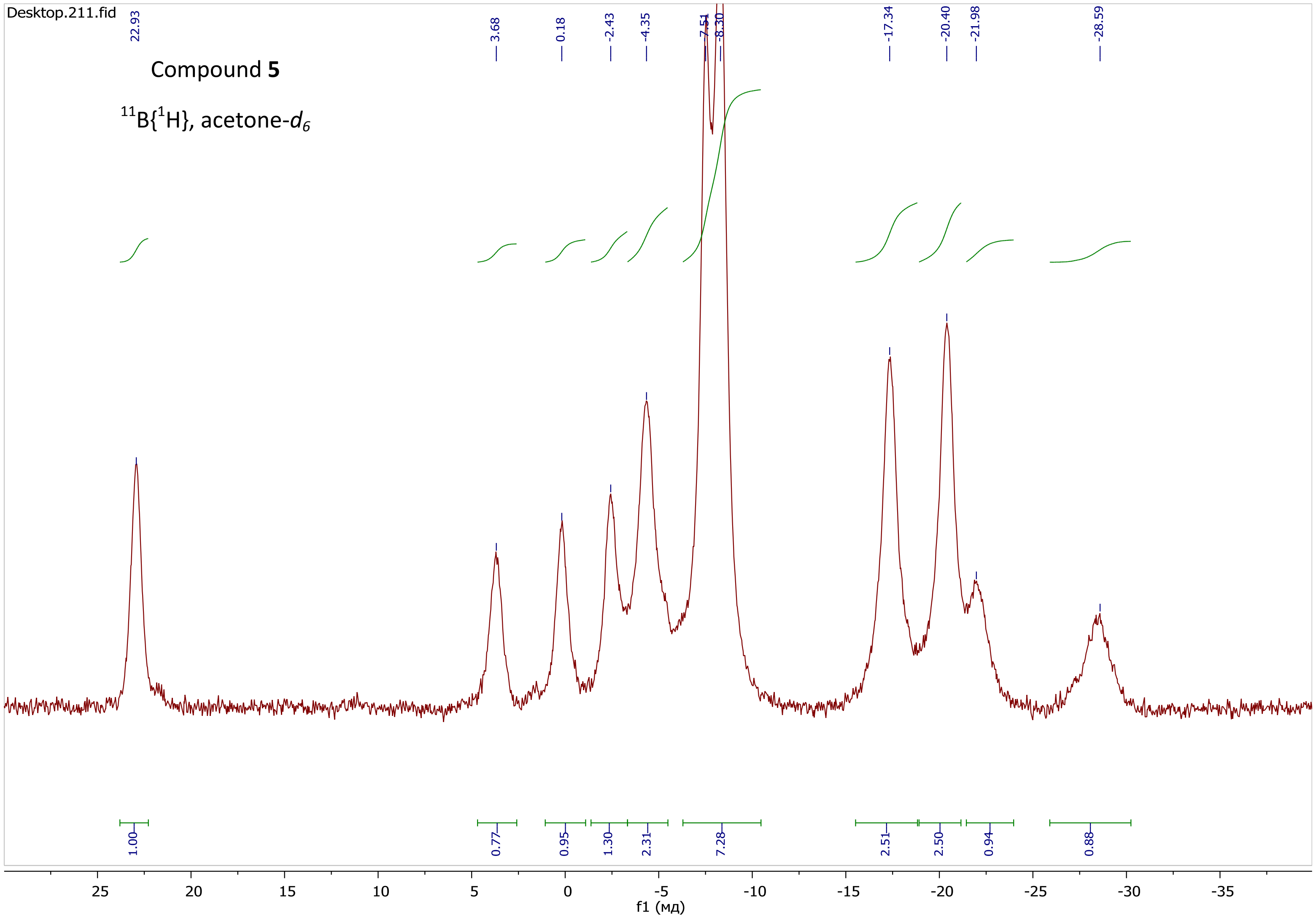

Figure S16. $^{11}B\{^{1}H\}$ NMR spectrum of compound **5**

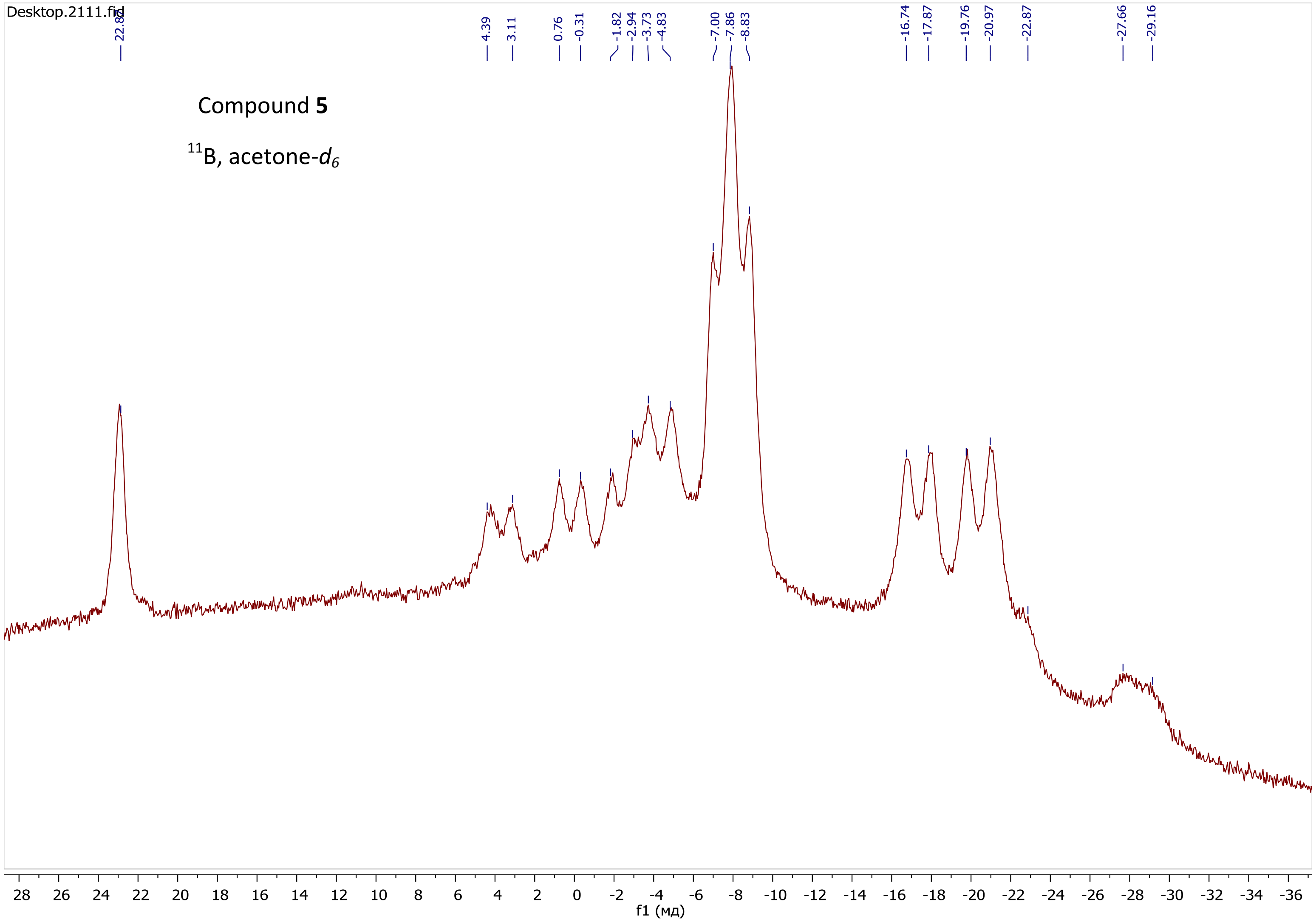

Figure S17. ^{11}B NMR spectrum of compound **5**

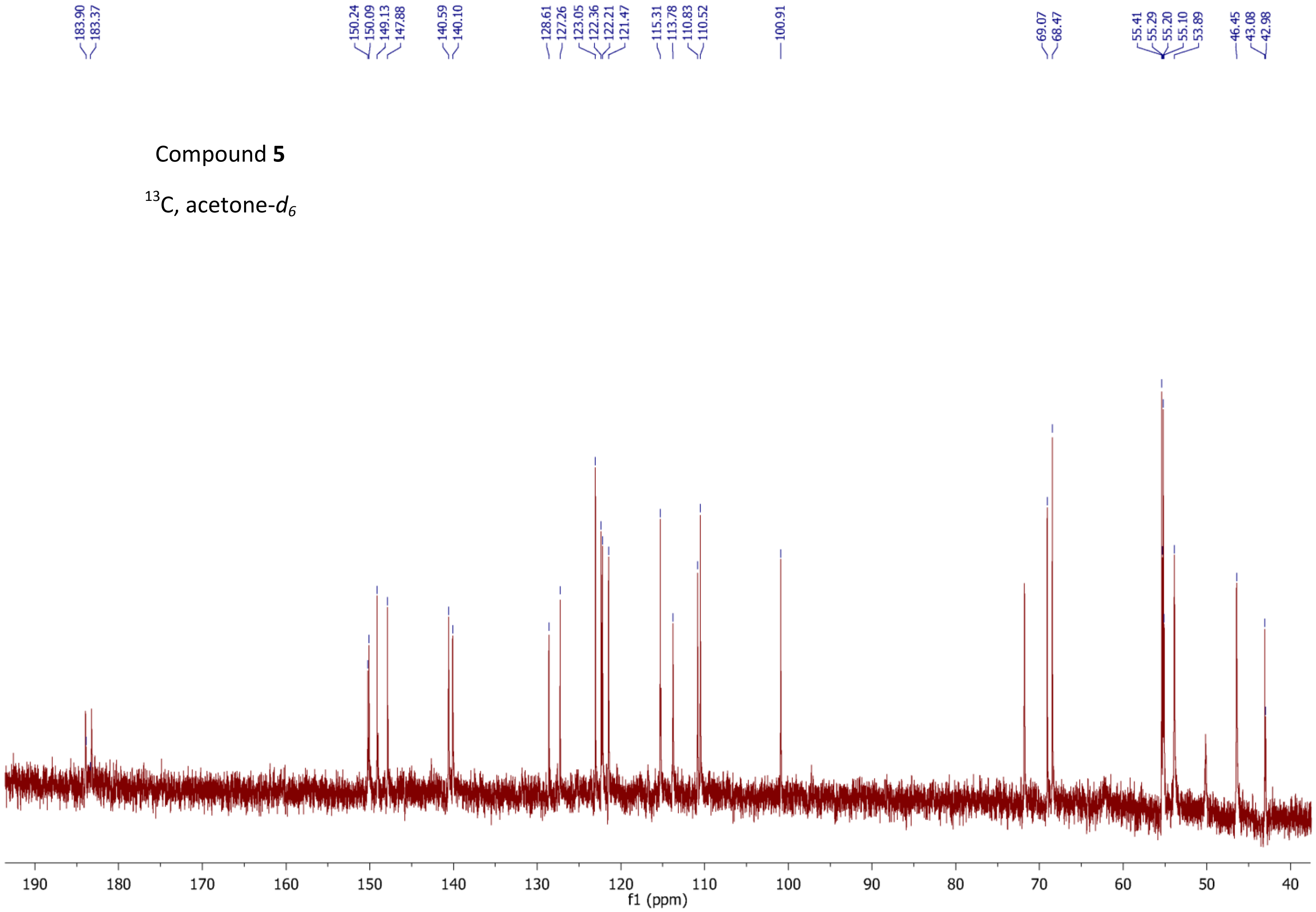

Figure S18. ^{13}C NMR spectrum of compound **5**

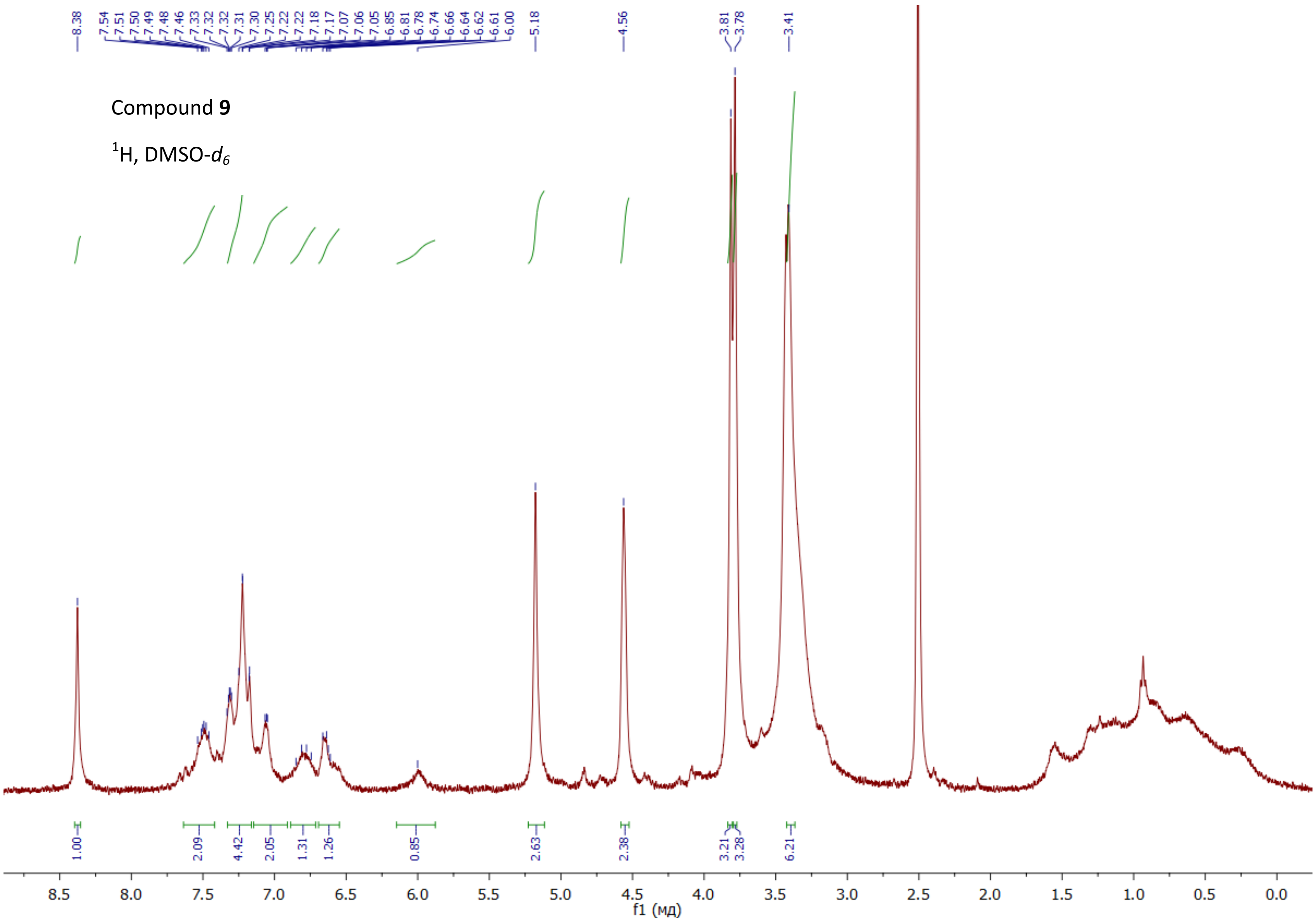

Figure S19. ^{1}H NMR spectrum of compound **9**

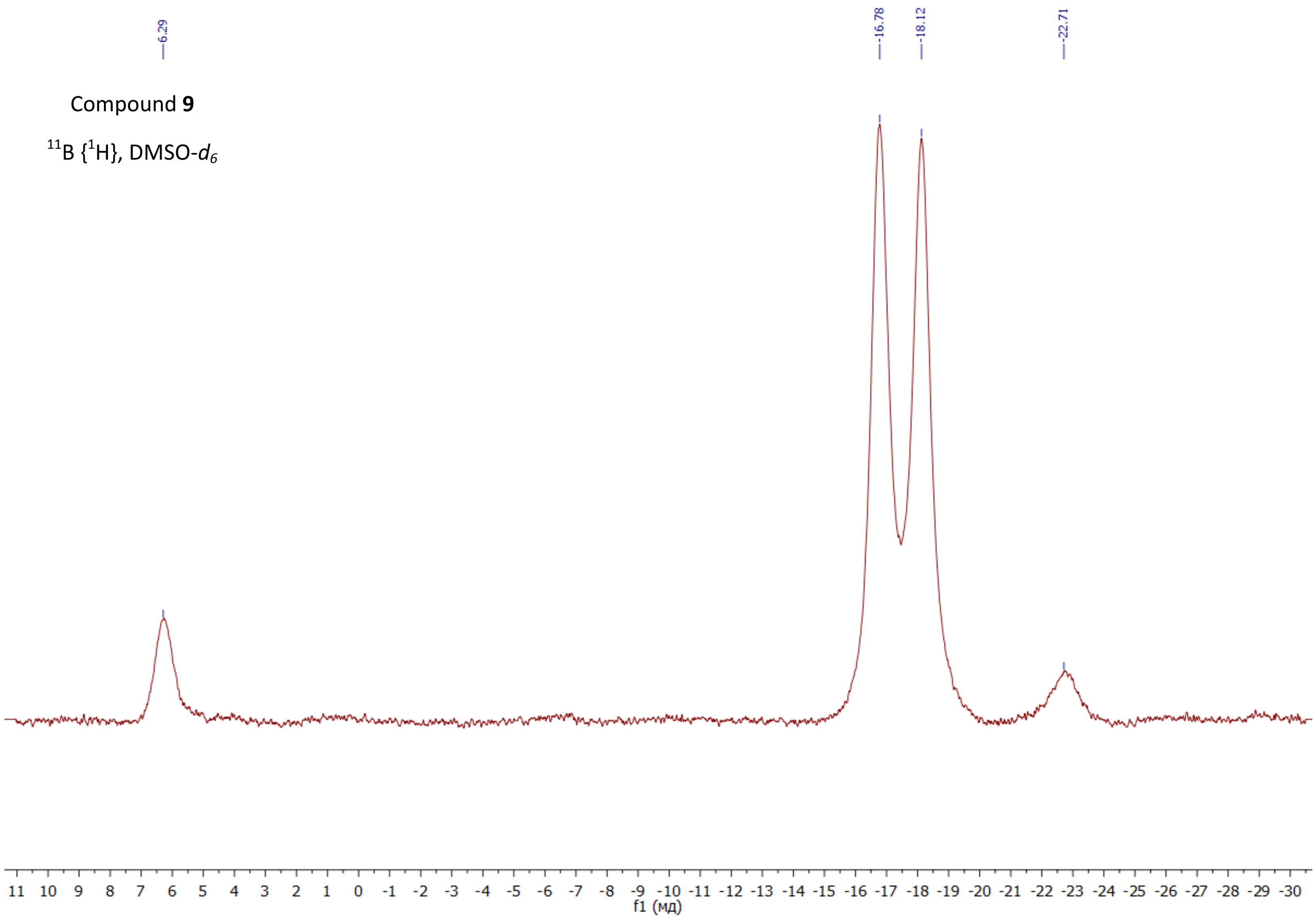

Figure S20. $^{11}B\{^1H\}$ NMR spectrum of compound **9**

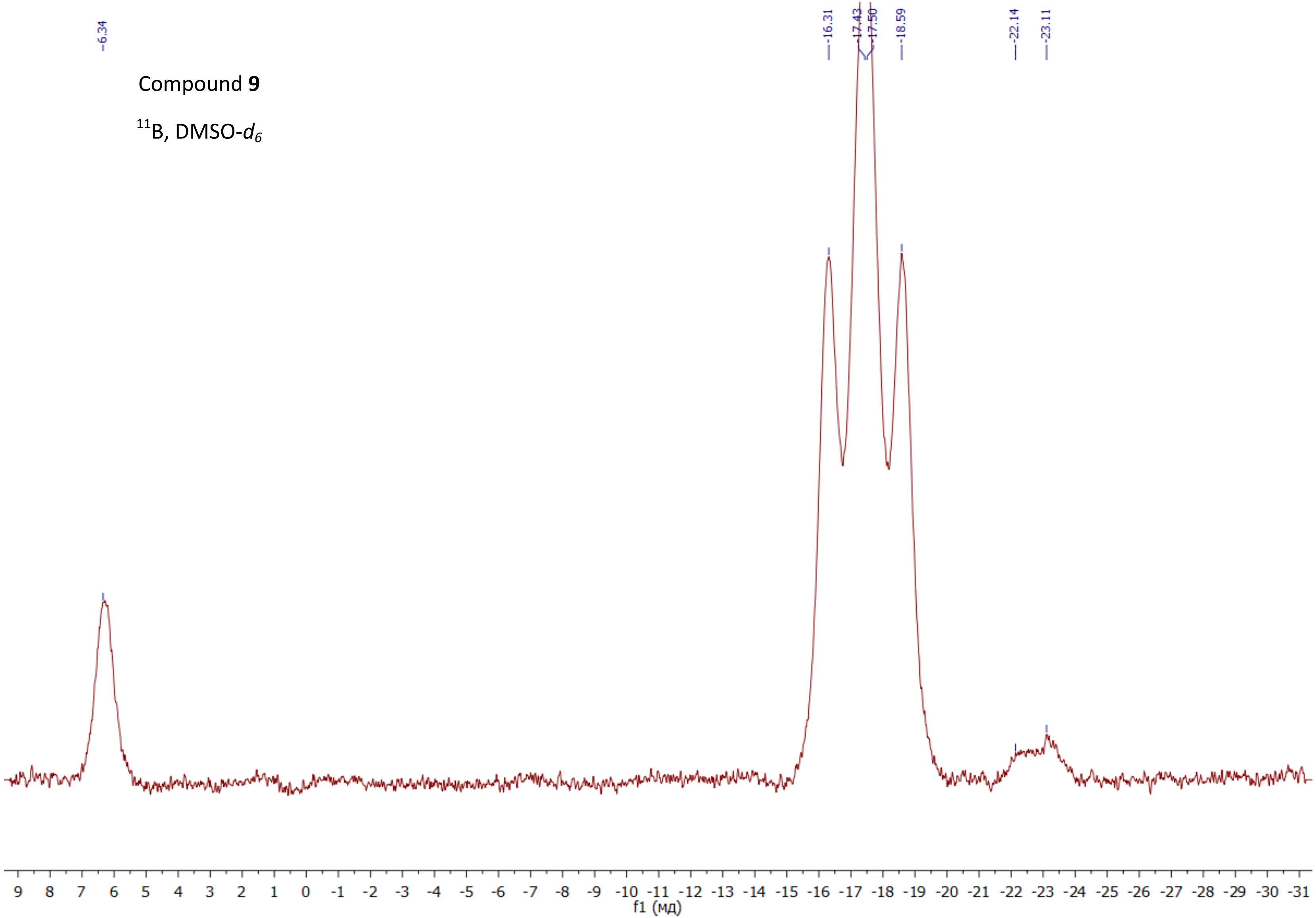

Figure S21. ^{11}B NMR spectrum of compound **9**

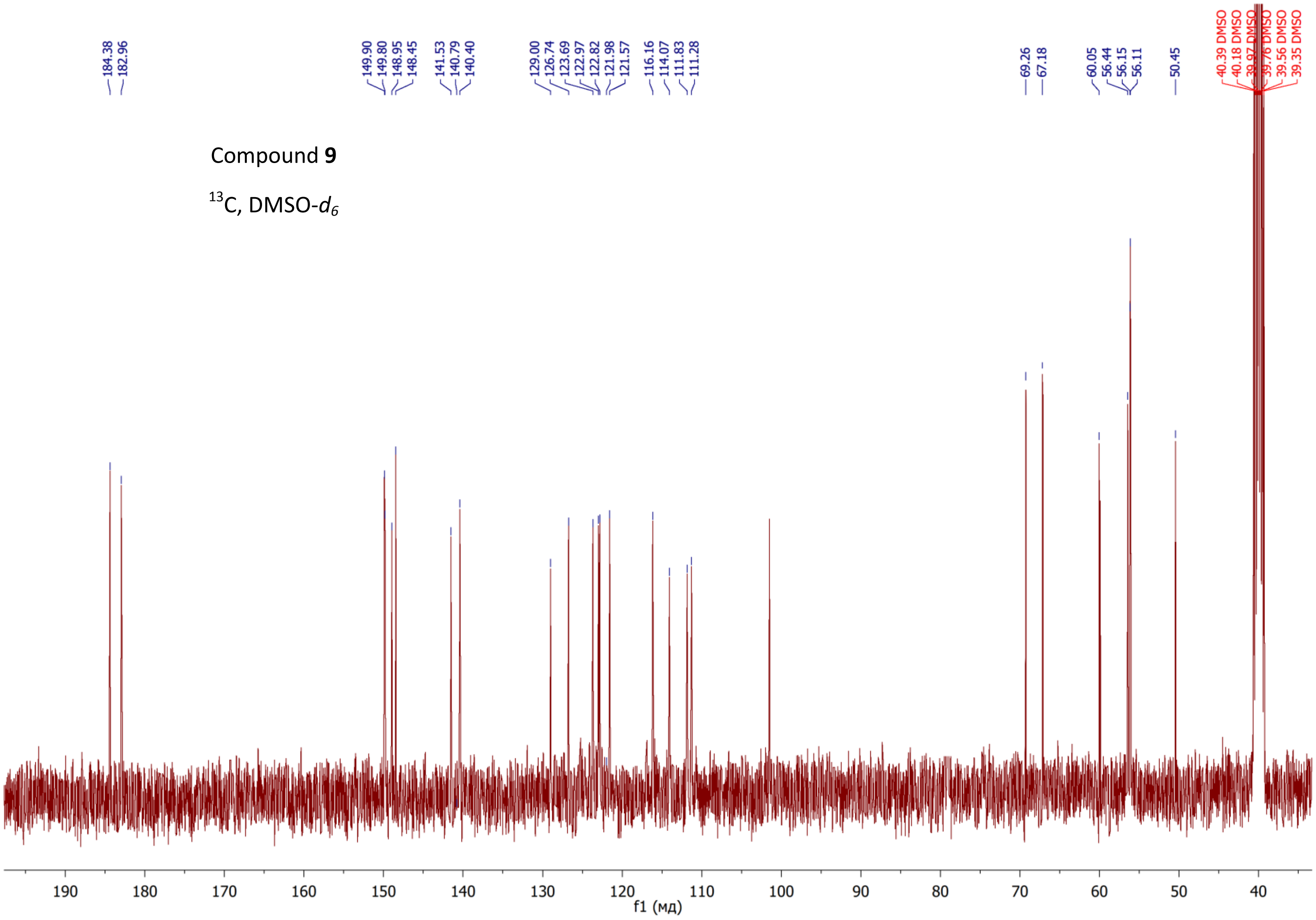

Figure S22. ^{13}C NMR spectrum of compound **9**

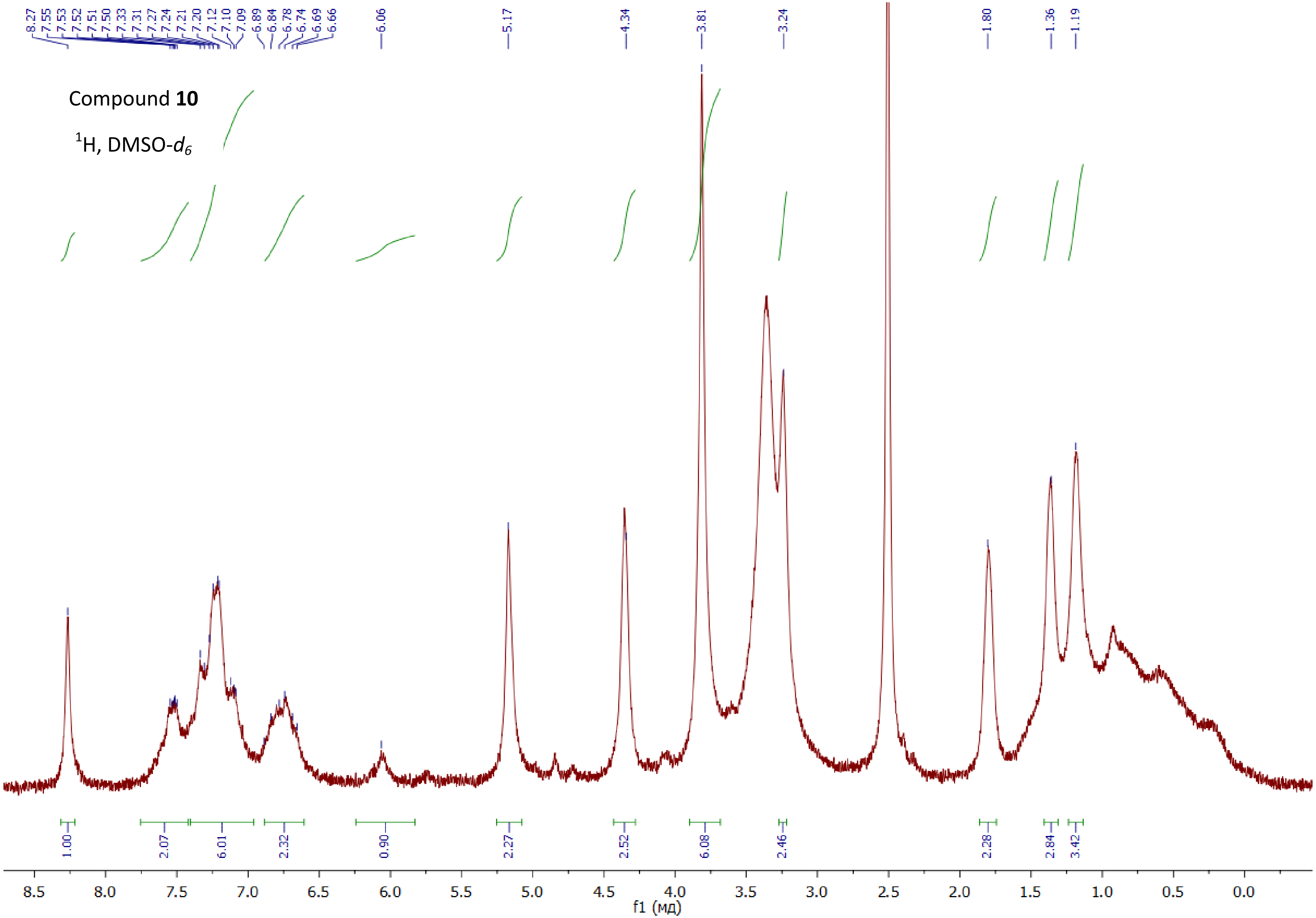

Figure S23. ^{1}H NMR spectrum of compound **10**

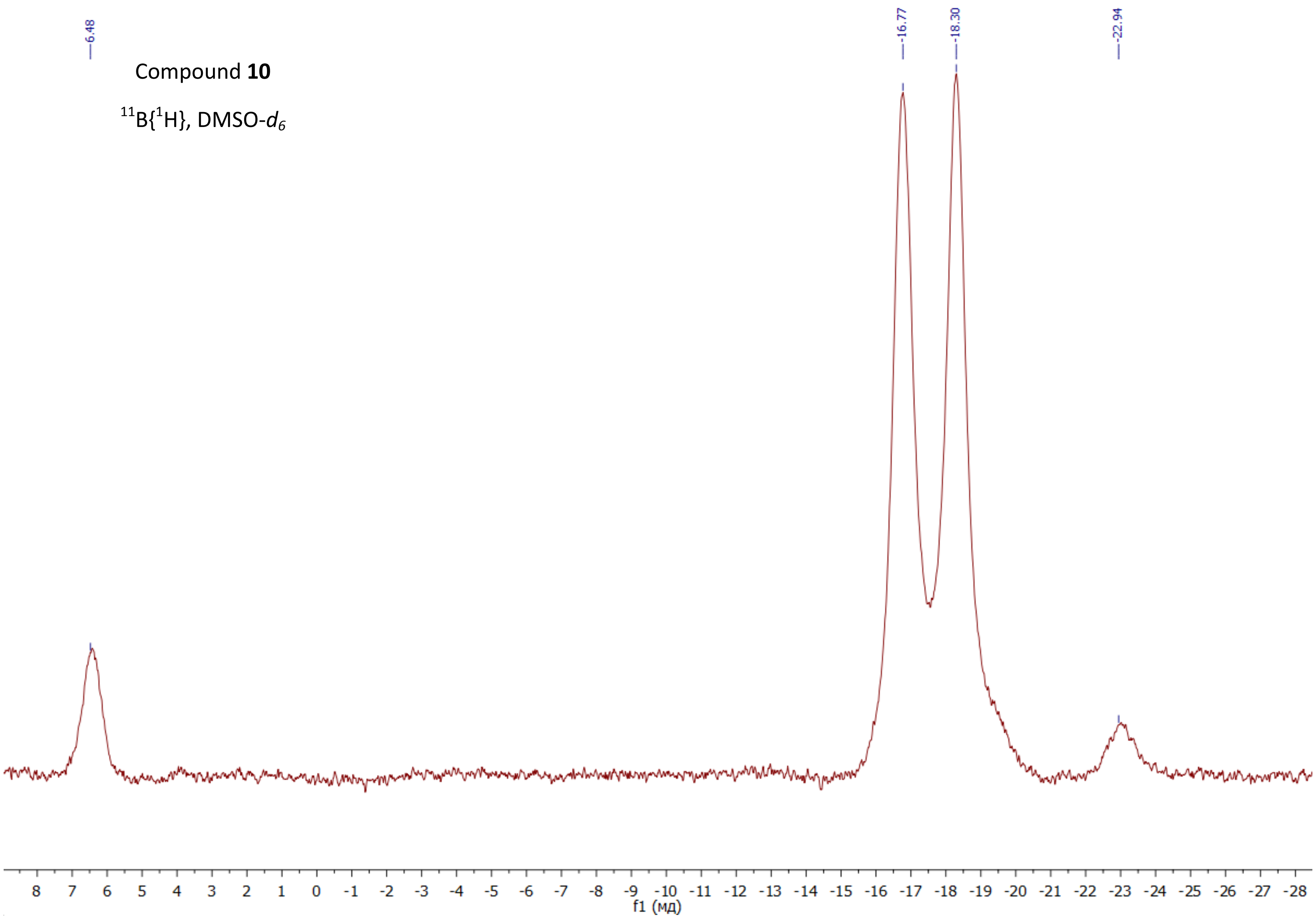

Figure S24. $^{11}B\{^1H\}$ NMR spectrum of compound **10**

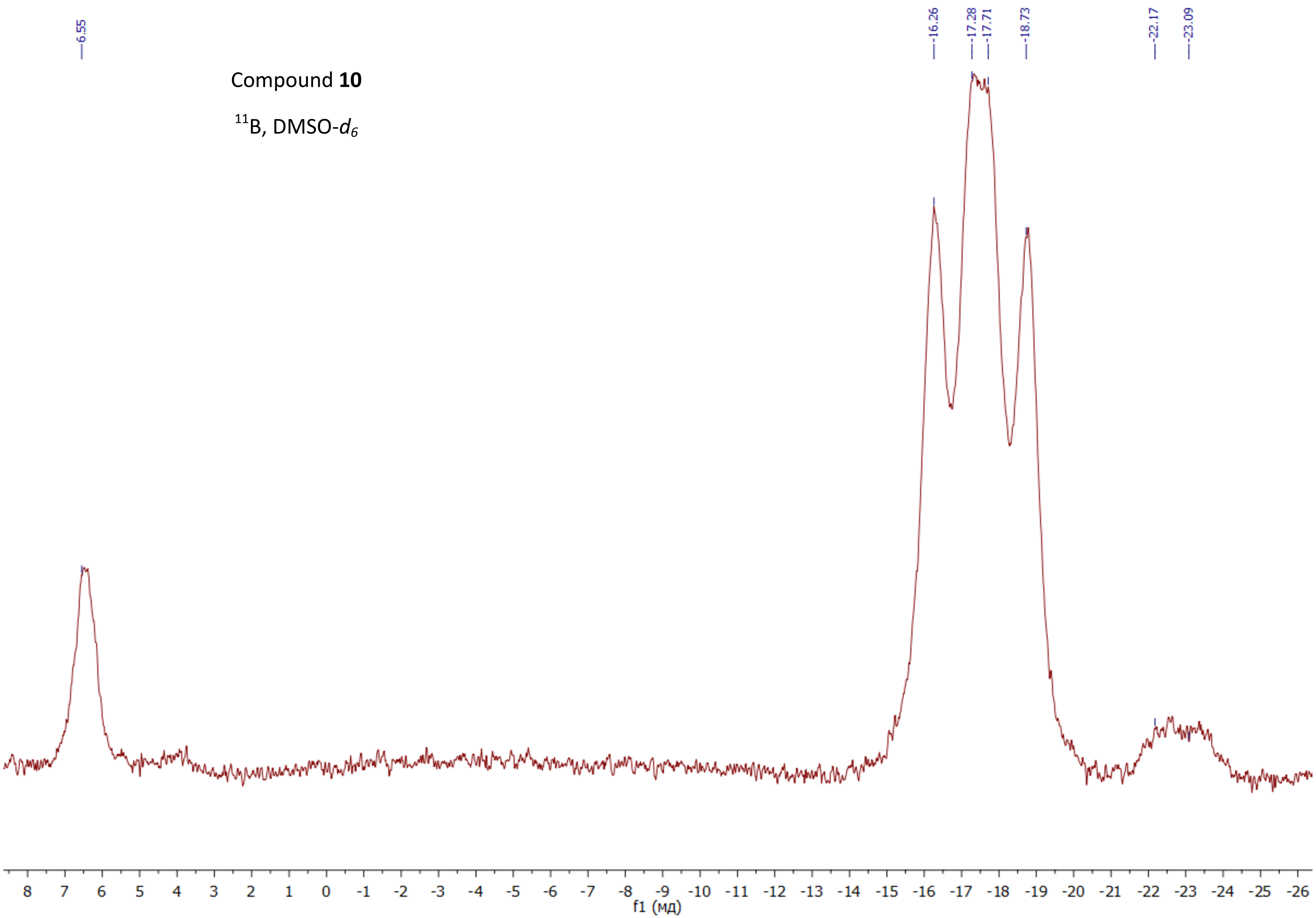

Figure S25. ^{11}B NMR spectrum of compound **10**

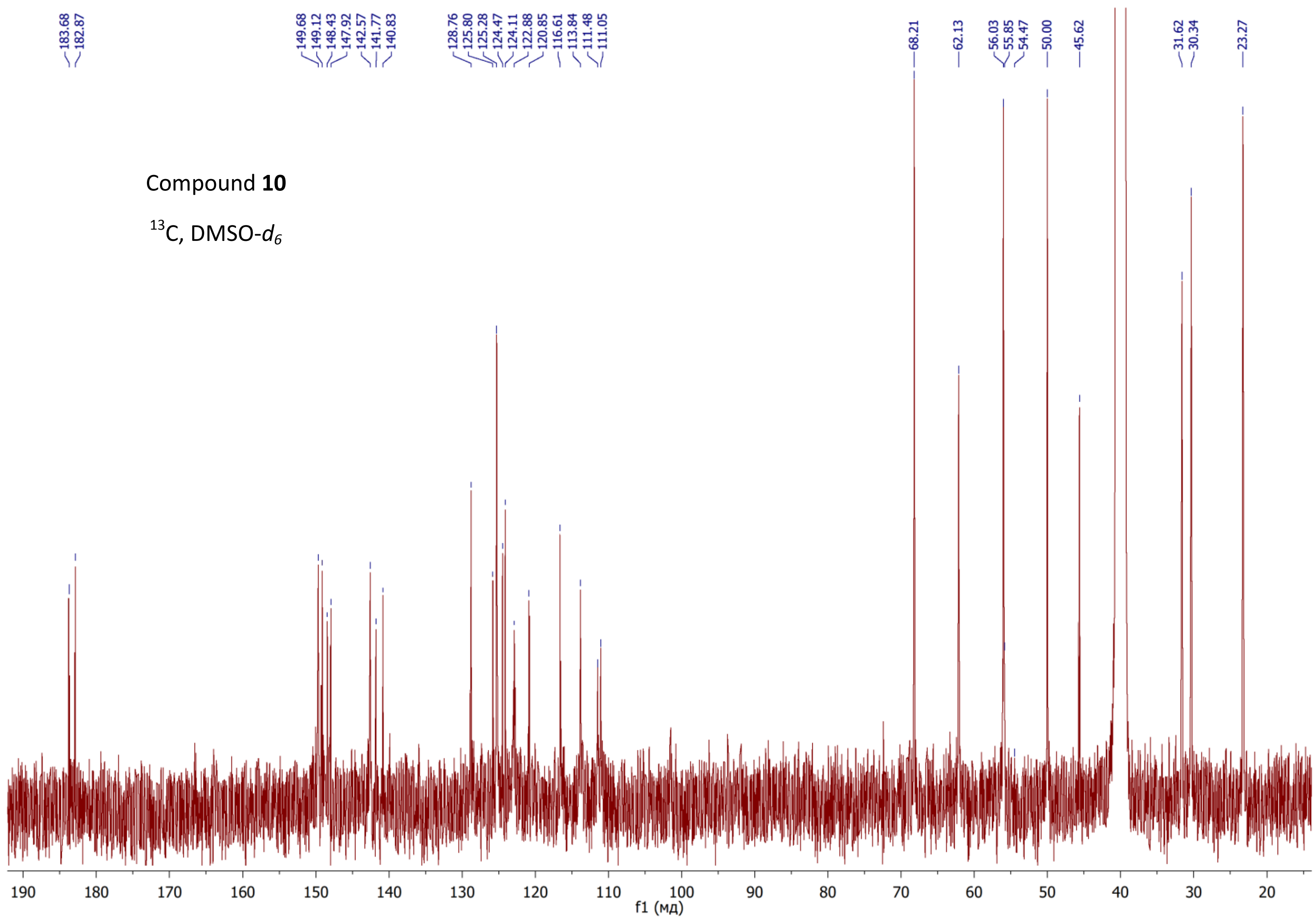

Figure S26. ^{13}C NMR spectrum of compound **10**

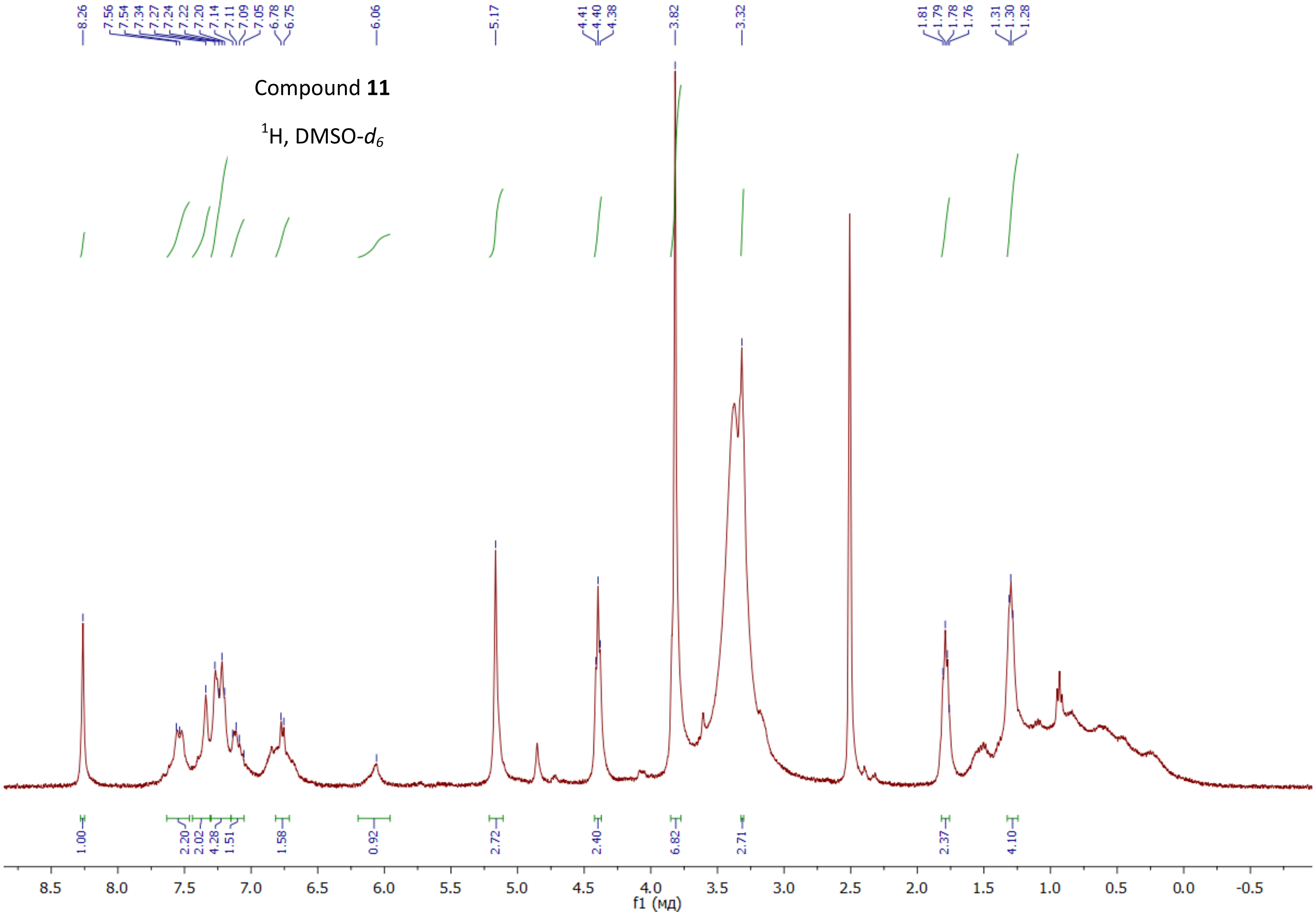

Figure S27. ¹H NMR spectrum of compound **11**

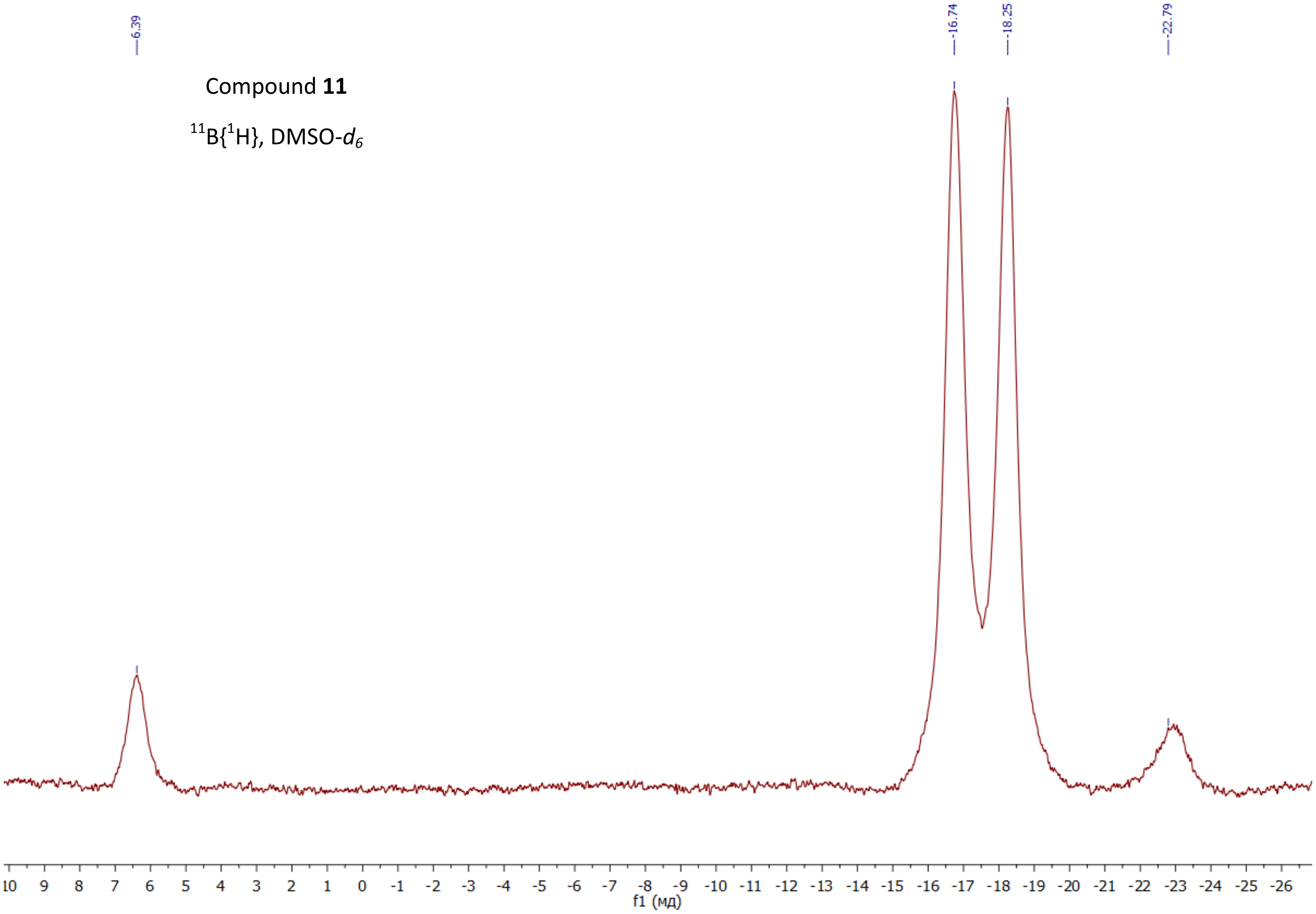

Figure S28. $^{11}B\{^1H\}$ NMR spectrum of compound **11**

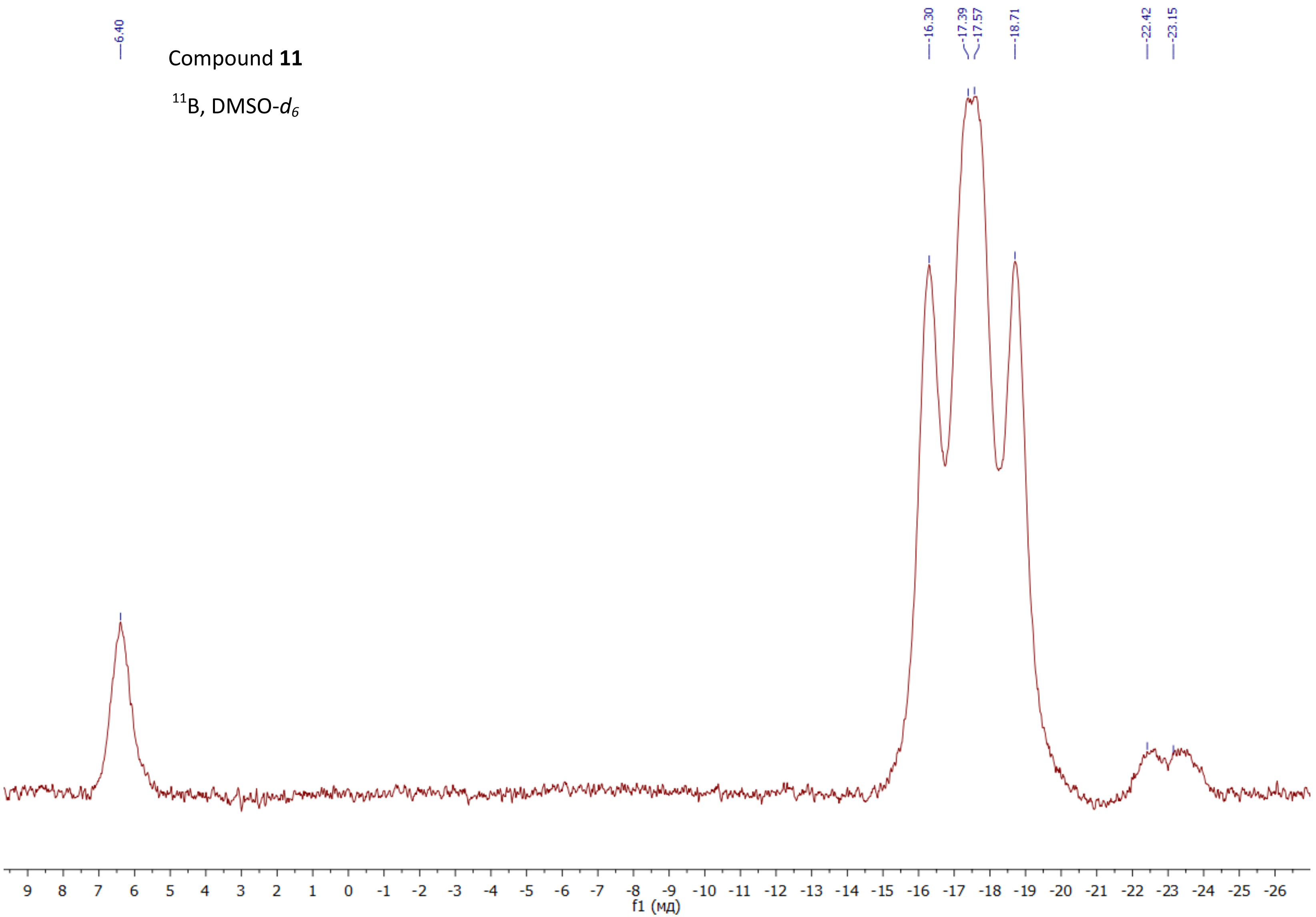

Figure S29. ^{11}B NMR spectrum of compound **11**

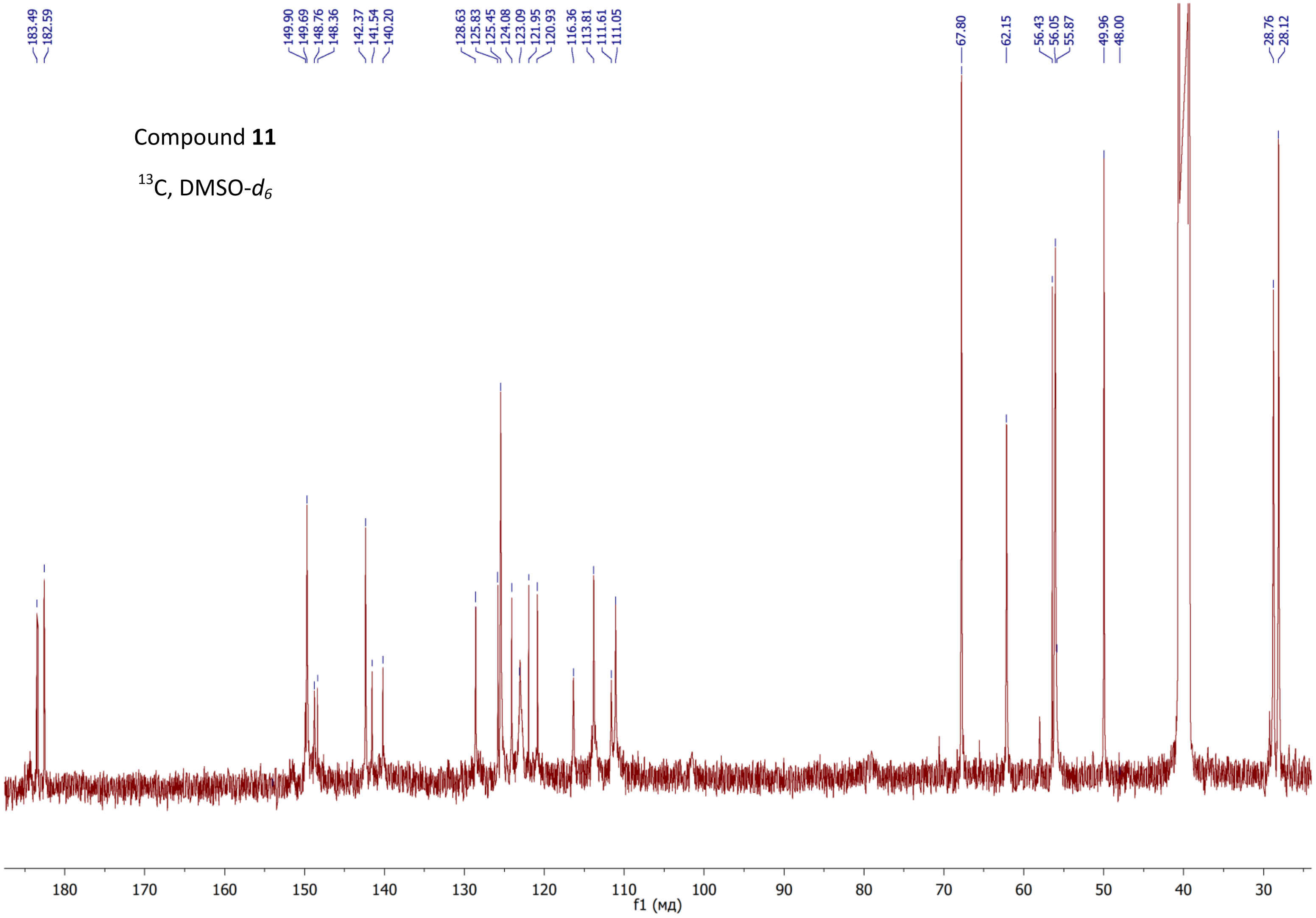

Figure S30. ^{13}C NMR spectrum of compound **11**
